# Non‐Statistical Assembly of Donor–Acceptor Cages for Light‐Induced Charge Separation

**DOI:** 10.1002/anie.202522944

**Published:** 2026-01-09

**Authors:** Jacopo Tessarolo, Laura Neukirch, Kai Wu, Jan‐Hendrik Borter, Haeri Lee, Dirk Schwarzer, Guido H. Clever

**Affiliations:** ^1^ Department of Chemistry and Chemical Biology TU Dortmund University Otto‐Hahn Straße 6 44227 Dortmund Germany; ^2^ Department of Chemistry Chonnam National University 77 Yongbong‐ro, Buk‐gu Gwangju 61186 Republic of Korea; ^3^ Lehn Institute of Functional Materials Institute of Green Chemistry and Molecular Engineering Guangdong Basic Research Center of Excellence for Functional Molecular Engineering MOE Key Laboratory of Bioinorganic and Synthetic Chemistry School of Chemistry Sun Yat‐Sen University Guangzhou 510275 China; ^4^ Department of Dynamics at Surfaces Max‐Planck‐Institute for Multidisciplinary Sciences Am Fassberg 11 37077 Göttingen Germany; ^5^ Department of Chemistry Hannam University Daejeon Republic of Korea; ^6^ Dortmund Center for Advanced Exploration of Dynamics Across Limits Using Spectroscopy (DAEDALUS) TU Dortmund University Otto‐Hahn‐Str. 4a 44227 Dortmund Germany

**Keywords:** Coordination cages, Electron transfer, Photoredox chemistry, Self‐assembly, Supramolecular chemistry

## Abstract

We report light‐triggered charge separation in two discrete supramolecular architectures that self‐assemble in a single step from donor (**D**) and acceptor (**A**) functionalized bridging ligands and Pd(II) cations. The “shape complementary assembly” (SCA) strategy allows for exclusive formation of the *cis*‐[Pd_2_ **D**_2_ **A**_2_]^4+^ cage isomer. Compared to previously reported statistical **DA** assemblies, lacking stoichiometry and stereo control, the number of possible electron transfer routes was reduced. This enables a better understanding and tunability of the excited state dynamics. Cage assembly was investigated by NMR, MS, and single crystal X‐ray diffraction analysis. Steady‐state absorption and electrochemical properties indicate that donor and acceptor moieties remain largely independent in the electronic ground state. Femtosecond pump‐probe spectroscopy in the visible and infrared was applied to compare the fate of photoexcited states for pure ligands, donor‐ and acceptor‐only assemblies, and the donor–acceptor heteroleptic cages. For the latter, ultrafast intracage ligand‐to‐ligand charge separation is followed by two back electron transfer pathways, occurring on timescales of hundreds of picoseconds and around one nanosecond, assignable to **D**/**A** ligands facing each other in *cis*‐ or *trans*‐position. Our work shows that non‐statistical modular self‐assembly can be used for the precise positioning of photoredox‐active components in defined distances on the nanoscale.

## Graphical abstract

Donor–acceptor functionalized cages [Pd_2_ **D**_2_ **A**_2_]^4+^ with defined stoichiometry and stereoconfiguration were assembled in a single step by employing the shape complementary assembly (SCA) strategy. While the redox functionalities remain largely independent in the ground state, photoexcitation results in ultrafast ligand‐to‐ligand charge transfer, as unraveled by spectro‐electrochemistry and transient absorption spectroscopy.

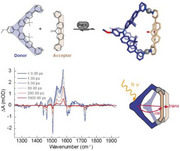

## Introduction

Sparked by a growing global energy demand, the depletion of fossil fuels, and the drawbacks associated with their combustion, a full transition towards renewable energy sources is inevitable.^[^ ^1^ ^]^ Amongst the different renewable sources, solar energy stands out owing to its wide availability. Commercially available photovoltaic systems are mostly based on silicon. While those systems are established and possess long‐term stability, they suffer from an energy‐intensive production and limited efficiency.^[^ ^2^, ^3^ ^]^ Nature has optimized its light‐harvesting apparatus over billions of years, taking a different approach: precisely arranged organic chromophores absorb sunlight and funnel electronic excitation to a reaction center where charge separation occurs.^[^ ^4^, ^5^ ^]^ Owing to favorable properties of organic chromophores, such as tunability, lightweight, and high absorption coefficients, the interest in organic photovoltaics is rising.^[^ ^6^, ^7^ ^]^ In this regard, a multitude of systems based on covalently connected electron donor and acceptor moieties has been investigated.^[^ ^8^, ^9^, ^10^, ^11^, ^12^ ^]^ This enabled drawing important structure‐property relationships such as the dependence of charge separated state lifetimes on the donor–acceptor spatial distance and their relative energetic alignment.^[^ ^13^, ^14^, ^15^ ^]^ However, the preparation of such covalent systems usually involves resource‐consuming multiple‐step organic synthesis and purification methods. Supramolecular chemistry represents an alternative to this shortcoming.^[^ ^16^, ^17^ ^]^ Multiple building blocks, equipped with suitable binding sites, are mixed to self‐assemble into larger structures, often in a single synthetic step. This modular approach allows for readily accessing libraries of systematically related derivatives and hence for rationally tuning properties of the photo(redox)‐active compounds. Coordination cages are a class of discrete self‐assembled supramolecules that are formed through the coordination of organic ligands to metal ions.^[^ ^18^, ^19^, ^20^ ^]^ While cages are mostly studied in the context of their host–guest chemistry, e.g. as catalysts^[^ ^21^, ^22^, ^23^ ^]^ or receptors,^[^ ^24^, ^25^, ^26^ ^]^ their formation has also been recognized as an efficient strategy for bringing multiple organic chromophores in close proximity in a single step.^[^ ^27^, ^28^, ^29^ ^]^

**Figure 1:**
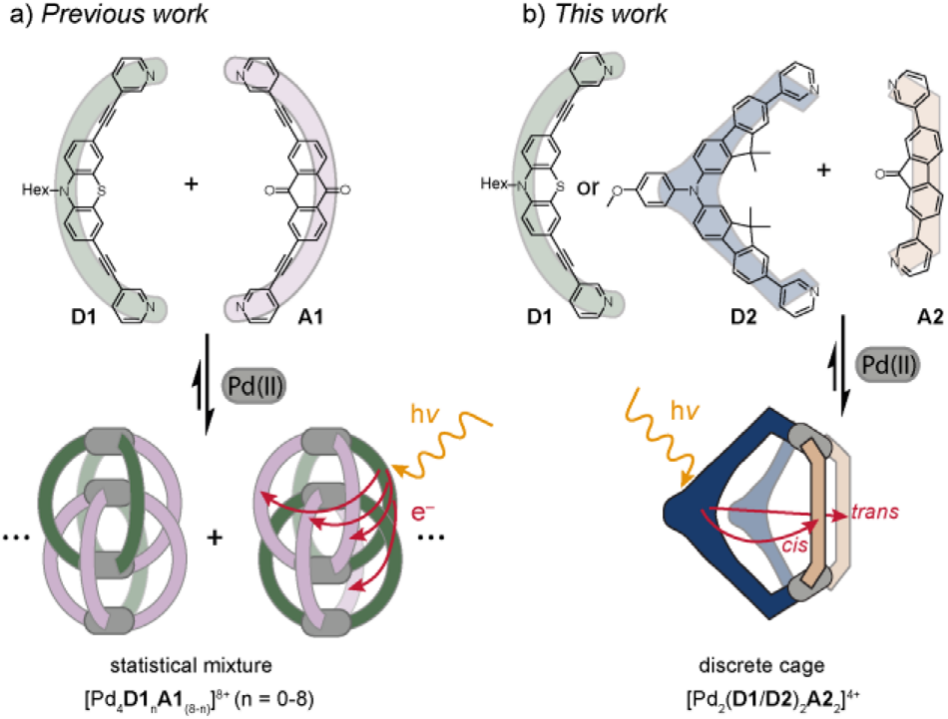
Development of Pd(II)‐based donor‐acceptor functionalized coordination cages: a) statistical approach to a mixture of mixed‐ligand interpenetrated dimers, b) herein introduced non‐statistical assembly of well‐defined *cis*‐[Pd_2_ **D**_2_ **A**_2_]^4+^ heteroleptic cages.

For example, Ward^[^ ^30^, ^31^ ^]^ and we^[^ ^32^ ^]^ reported on the photoinduced charge transfer from electron‐rich cages to encapsulated acceptor guests. While host‐to‐guest electron transfer is interesting in the context of photoredox catalysis, this donor–acceptor arrangement with one component enclosed by the other may hamper further separation of charges in opposite directions as required for photovoltaic materials. Therefore, the investigation of ligand‐to‐ligand electron transfer in mixed‐ligand assemblies moved into focus. In previous work in this direction, we prepared statistical mixtures of interlocked double cages [Pd_4_ **D1** *_n_* **A1**_(8−_ *_n_*_)_]^8+^ (*n* = 0 8) based on Pd(II) metal nodes, phenothiazine‐based donor ligand **D1**, and anthraquinone‐based acceptor ligand **A1** (Figure 1a).^[^ ^33^, ^34^ ^]^ We showed that those cages undergo photoinduced charge separation within 200 fs to yield species [Pd_4_(**D1^•+^**)(**D1**_(_ *_n_*_−1)_)(**A1^•−^**)(**A1**_(7−n)_)]^8+^. Charge recombination occurs then along multiple pathways with timescales ranging from picoseconds to nanoseconds. Owing to the statistical distribution of donor and acceptor ligands within the cage, a multitude of electron transfer pathways is feasible. This hampers a clear assignment of time constants and hence drawing structure‐function relationships, crucial for fully understanding and optimizing the system. During the last decade, we and others introduced several strategies to control the integrative self‐sorting of various functionalities into defined mixed‐ligand (heteroleptic) coordination cages.^[^ ^20^, ^35^, ^36^ ^]^ For example, the “coordination sphere engineering” (CSE) strategy exploits steric bulk right on the donor sites to steer integrative self‐assembly into heteroleptic cages with full control over ligand stoichiometry and positioning.^[^ ^37^, ^38^ ^]^ Other groups exploited cavity‐centered steric bulk for driving heteroleptic cage formation.^[^ ^39^, ^40^ ^]^ Another approach uses geometric constraints: a convergent bridging ligand **1** is combined with a divergent ligand **2**. Heteroleptic cage *cis*‐[Pd_2_ **1**_2_ **2**_2_]^4+^ is then favored over the homoleptic counterparts from an enthalpic point of view (reduced conformational strain) as well as from an entropic perspective (low nuclearity).^[^ ^41^, ^42^ ^]^ We have applied this “shape complementary assembly” (SCA) approach for generating a variety of functional cages.^[^ ^43^, ^44^ ^]^ For example, the combination of chiral with fluorescent ligands allowed for the observation of circularly polarized luminescence from an initially non‐chiral ligand.^[^ ^45^, ^46^ ^]^ In order to achieve the highest level of self‐sorting in this type of lantern‐shaped dinuclear palladium cages—the formation of a single [Pd_2_ **1**_1_ **2**_1_ **3**_1_ **4**_1_]^4+^ isomer—we recently combined the SCA approach with a further strategy based on “adjacent backbone interactions” (ABI).^[^ ^47^ ^]^

Herein, we use the SCA approach to access two discrete donor–acceptor functionalized *cis*‐[Pd_2_ **D**_2_ **A**_2_]^4+^ cages for the first time. The structural, photophysical, and electrochemical properties of the novel supramolecular structures are thoroughly analyzed. The fate of the photoexcited state is unraveled by transient absorption spectroscopy in the visible and infrared probe region. The defined distances between donor and acceptor ligands in the integratively self‐sorted cages enable a more profound understanding of the light‐induced dynamics.

## Results and Discussion

### Design and Synthesis

**Figure 2:**
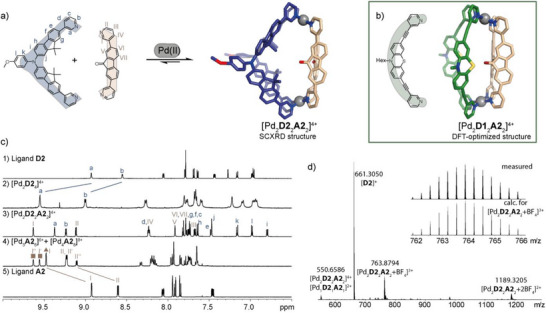
a) Synthesis of donor–acceptor cages via the SCA strategy and SCXRD structure of [Pd_2_ **D2**_2_ **A2**_2_]^4+^; b) DFT‐model of [Pd_2_ **D1**_2_ **A2**_2_]^4+^ (B3LYP/Def2‐SVP; hexyl chains substituted by methyl groups); c) Aromatic region of ^1^H NMR spectra (500 or 700 MHz, CD_3_CN, 298 K) of ligands, homoleptic assemblies, and heteroleptic cage [Pd_2_ **D2**_2_ **A2**_2_]^4+^ (some signals of 3‐ and 4‐membered species in spectrum 4 assigned by triangle/square symbols); d) HR‐ESI‐MS of [Pd_2_ **D2**_2_ **A2**_2_]^4+^, the inset shows the measured and calculated isotopic pattern of [Pd_2_ **D2**_2_ **A2**_2 _+ BF_4_]^3+^.

Electron‐rich donor and electron‐poor acceptor ligands were designed so they possess complementary geometries to obtain discrete cages *cis*‐[Pd_2_ **D**_2_ **A**_2_]^4+^ via the SCA approach. For the first combination, triarylamine and fluorenone, serving as well‐known donor^[^ ^11^ ^]^ and acceptor^[^ ^48^ ^]^ functionalities, were chosen as backbones. Ligand **D2**, based on triarylamine, features convergent donor vectors (*α* = −34°) and an N···N distance of 14.1 Å. Fluorenone‐based ligand **A2** is approximately to the same extent divergent as ligand **D2** is convergent (*α* = +36°) with a slightly smaller N···N distance of 12.6 Å, as required for a *cis*‐[Pd_2_A_2_B_2_]^4+^‐type cage with non‐coplanar Pd(py)_4_ units (Figure 2a).

**Figure 3:**
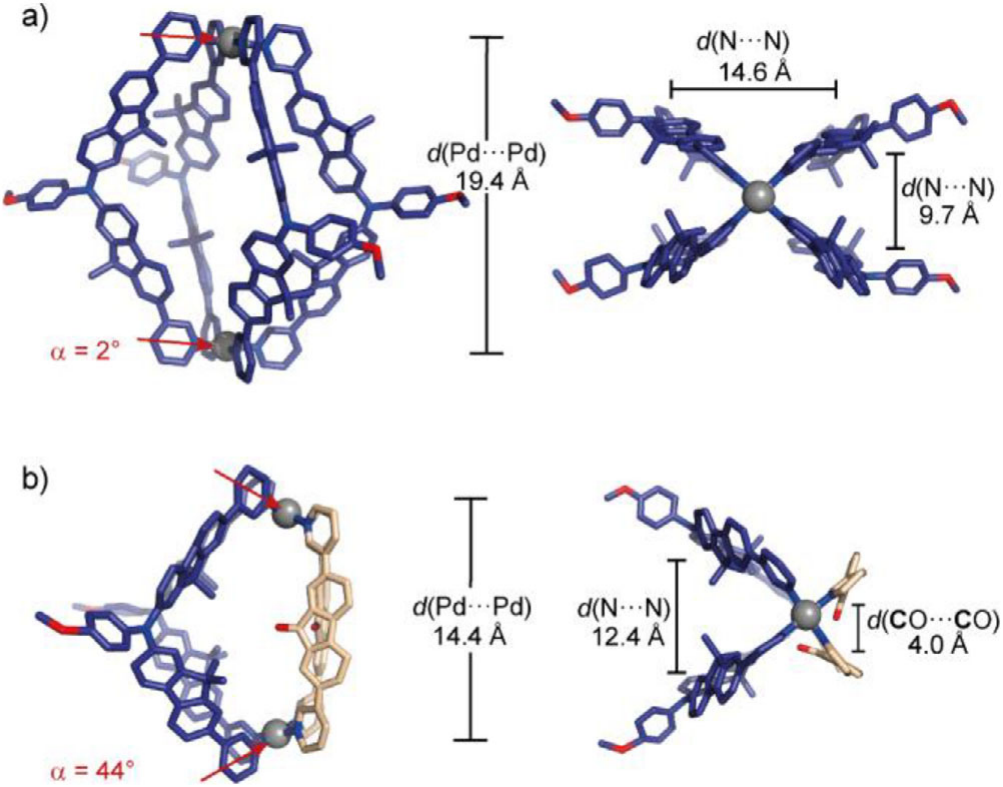
X‐ray crystal structures of a) homoleptic [Pd_2_ **D2**_4_]^4+^ (left: side view, right: top view) and b) donor–acceptor cage [Pd_2_ **D2**_2_ **A2**_2_]^4+^ (left: side view, right: top view, α denotes the binding angle of ligand **D2**, for determination see Supporting Information, section 6.1).

Donor ligand **D2** was synthesized via Suzuki cross coupling followed by a Buchwald–Hartwig amination (Fig. S1‐S9). Acceptor ligand **A2** was synthesized in one step as reported by us beforehand.^[^ ^49^ ^]^ First, the homoleptic assembly of new ligand **D2** was investigated. Self‐assembly with [Pd(CH_3_CN)_4_](BF_4_)_2_ in DMSO‐*d*_6_ leads to a downfield shift of the signals of protons a and b that are close to the pyridine nitrogen, indicative for coordination to Pd(II). ^1^H DOSY NMR yielded a hydrodynamic radius of 12.6 Å, suggesting formation of a lantern‐shaped cage. In accordance with this, the formula [Pd_2_ **D2**_4_]^4+^ was obtained by high‐resolution electrospray ionization mass spectrometry (HR‐ESI‐MS). Slow vapor diffusion of benzene into a solution of the assembly in DMSO yielded yellow, needle‐shaped single crystals suitable for synchrotron diffraction analysis. The compound crystallizes in space group P‐3 with half of the cage in the asymmetric unit. In the lantern‐shaped cage, the centers of the backbones form a rectangle (rather than a square as expected for a *D*_4h_‐symmetric cage); more precisely, two adjacent backbones are either positioned in a distance of 14.6 Å or 9.7 Å, respectively (measured between the backbone nitrogen atoms, Figure 3a).

In the solid‐state packing, the ligand of one cage is stacked in between two ligands of a neighboring cage, leading to this distortion. Compared to the pyridine N···N distance of the flat ligand, cage [Pd_2_ **D2**_4_]^4+^ is elongated along the Pd‐Pd axis (*d*(Pd···Pd) = 19.4 Å), showcasing the conformational flexibility of ligand **D2**. In this conformation, the donor vectors are nearly collinear (*α* = +2°), allowing for the formation of a homoleptic lantern‐shaped cage without a helical twist. Another lantern‐shaped cage Pd_2_L_4_ with triarylamine‐based ligands was recently reported by Yang and coworkers.^[^ ^50^ ^]^ On the other hand, ligand **A2** assembles into a three‐membered ring [Pd_3_ **A2**_6_]^6+^ in DMSO‐*d*_6_, while a mixture of ring [Pd_3_ **A2**_6_]^6+^ and pseudo‐tetrahedral [Pd_4_ **A2**_8_]^8+^ is yielded in CD_3_CN (Figure 2b), as shown by us before (note that the signals corresponding to the pseudo‐tetrahedron experience a two‐fold split owing to singly‐ and doubly‐bridged edges).^[^ ^49^ ^]^

Next, we combined ligands **D2** and **A2** with [Pd(CH_3_CN)_4_](BF_4_)_2_ in a 1:1:1 ratio in DMSO‐*d*_6_ or CD_3_CN. In both solvents, the ^1^H NMR spectrum shows a single set of signals, distinguishable from the signals of ligands **D2** and **A2** as well as the corresponding homoleptic assemblies, suggesting that a new species was obtained. Formation of heteroleptic cage [Pd_2_ **D2**_2_ **A2**_2_]^4+^ was confirmed by ^1^H DOSY NMR (*r*_H_  =  10.29 Å in CD_3_CN) and HR‐ESI‐MS. Moreover, needle‐shaped crystals suitable for synchrotron diffraction analysis were grown by slow vapor diffusion of benzene into a solution of the cage in DMSO. The compound crystallizes in space group P‐1 with an entire molecule in the asymmetric unit. In the crystal structure, the two pairs of ligands are arranged in a *cis*‐configuration and the PdN_4_ planes are tilted, characteristic for the formation of *cis*‐[Pd_2_A_2_B_2_]^4+^‐type cages via the SCA approach. As compared to homoleptic [Pd_2_ **D2**_4_]^4+^, the cage is shortened along its Pd‐Pd axis (*d*(Pd···Pd) = 14.4 Å) and the pyridine N···N distances as well as the bent angle of ligand **D2** resemble much closer the ones in the unstrained, free ligands (*d*(N···N, **D2**) = 15.9 Å; α(**D2**) = +44°; *d*(N···N, **A2**) = 13.1 Å), indicating a perfect structural match between the ligands in the heteroleptic assembly.

We also tested the heteroleptic self‐assembly of acceptor ligand **A2** with formerly reported donor ligand **D1**. While the combination of phenothiazine and fluorenone was expected to be promising for achieving photoinduced charge separation,^[^ ^51^ ^]^ we suspected that a clean self‐assembly would be hampered by the nearly collinear binding vectors of ligand **D1** (*α* = −5°).^[^ ^33^ ^]^ However, the combination of ligands **D1**, **A2**, and [Pd(CH_3_CN)_4_](BF_4_)_2_ in a 1:1:1 ratio in CD_3_CN likewise cleanly yielded a discrete species, as attested by the single set of signals in the ^1^H NMR spectrum (Figure S22). The ^1^H DOSY NMR spectrum as well as the ESI mass spectrum furthermore suggest that in analogy to the outcome with donor ligand **D2**, *cis*‐[Pd_2_ **D1**_2_ **A2**_2_]^4+^ had formed exclusively. In the DFT‐computed model of the cage (B3LYP/Def2‐SVP), the alkyne linkers of ligand **D1** bend, enabling adoption of a conformation with convergent binding vectors, which is shape complementary to the divergent fluorenone ligand (*d*(N···N, **D1**) = 15.5 Å; *α*(**D1**) = +24°; *d*(N···N, **A2**) = 13.4 Å). Similar observations were made by the groups of Severin^[^ ^52^ ^]^ and Lewis^[^ ^53^ ^]^ for cages assembled from comparable ligands with collinear binding vectors.

### Steady‐State Optical Spectroscopy

**Figure 4:**
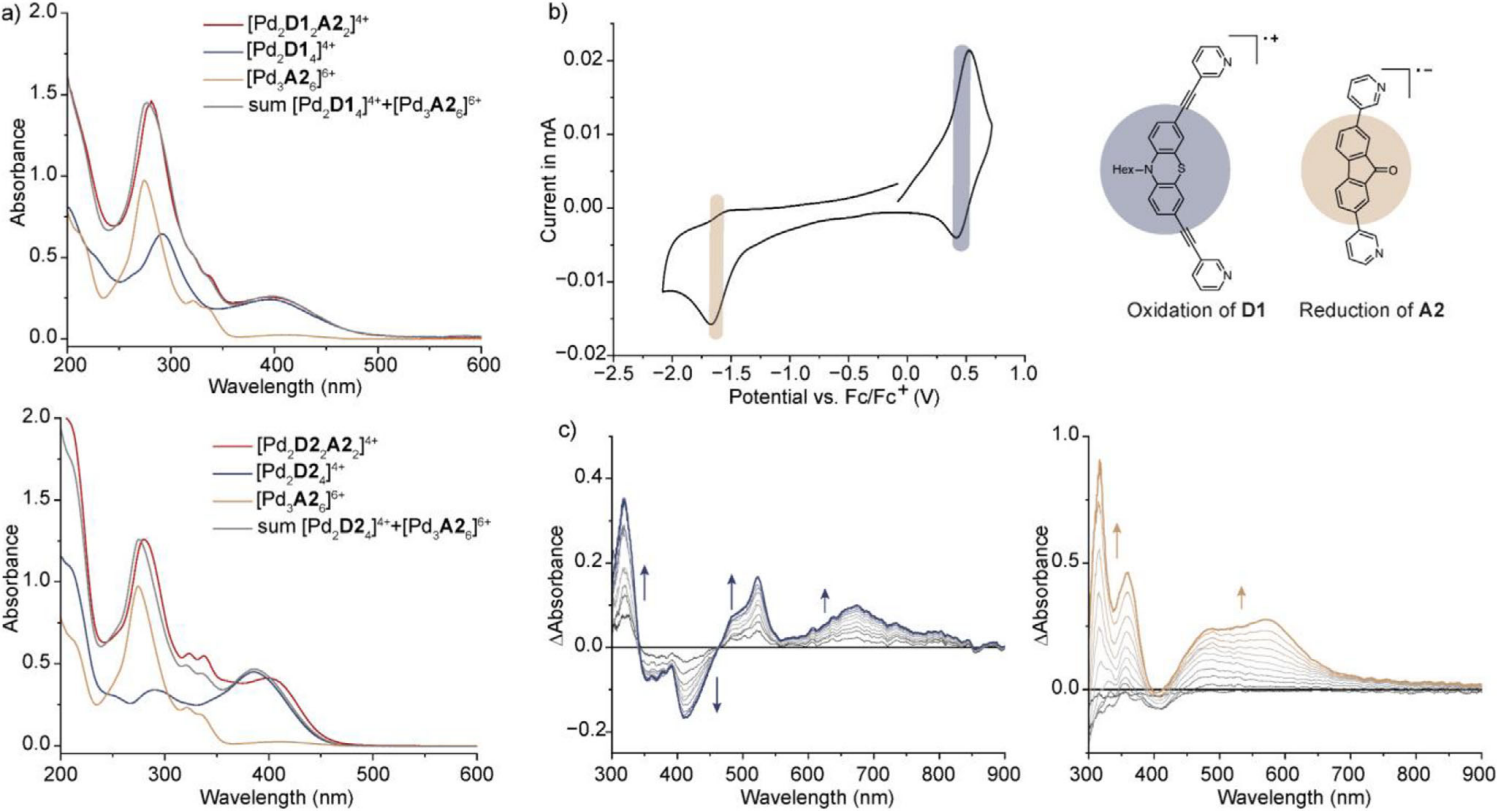
a) UV–vis absorption spectra of donor–acceptor cages, the corresponding homoleptic assemblies and the calculated sum thereof for [Pd_2_ **D1**_2_ **A2**_2_]^4+^ (top) and [Pd_2_ **D2**_2_ **A2**_2_]^4+^ (bottom, *c*  =  50 µM, CH_3_CN, 1 mm cuvette pathlength); b) cyclic voltammogram of [Pd_2_ **D1**_2_ **A2**_2_]^4+^ (*c*  =  160 µM, CH_3_CN, 0.1 M TBAPF_6_, working electrode (WE): glassy carbon, counter electrode (CE): platinum wire, reference electrode (RE): Ag/AgNO_3_, scan rate: 0.1 V·s^−1^); c) UV–vis difference spectra upon oxidation (left) and reduction (right) of [Pd_2_ **D1**_2_ **A2**_2_]^4+^ (*c*  =  160 µM, CH_3_CN, 0.1 M TBAPF_6_, WE: gold gauze, CE: platinum wire, RE: Ag/AgNO_3_, 0.5 mm cuvette pathlength).

Next, the photophysical properties of the donor–acceptor cages in CH_3_CN were studied and compared to the ones of the corresponding homoleptic assemblies. The spectrum of heteroleptic cage [Pd_2_ **D1**_2_ **A2**_2_]^4+^ shows absorption maxima at 282 and 400 nm as well as two less intense features at 323 and 336 nm (Figure 4a, top). The spectrum of the donor–acceptor cage is in very good accordance with the sum of the spectra of the corresponding homoleptic assemblies, indicating the absence of strong electronic communication between the ligands in their electronic ground states. Donor ligand **D1** assembles in CH_3_CN into interlocked double cage [Pd_4_ **D1**_8_]^8+^ as thermodynamic product.^[^ ^54^ ^]^ Owing to the dense packing of the ligands in this catenated structure, close inter‐ligand interactions are highly likely.^[^ ^33^ ^]^ For better comparability with the ligand environments in the less densely packed heteroleptic cages, we therefore considered here the spectrum of the kinetically stable non‐catenated monomeric cage [Pd_2_ **D1**_4_]^4+^. The spectrum of donor‐acceptor cage [Pd_2_ **D2**_2_ **A2**_2_]^4+^ possesses two maxima at 280 and 403 nm and, similarly to [Pd_2_ **D1**_2_ **A2**_2_]^4+^, two smaller features at 323 and 337 nm stemming from the acceptor **A2** (Figure 4a, bottom). In contrast to the system with the **D1** donor, the spectrum of the donor–acceptor cage with **D2** is redshifted by around 20 nm as compared to the sum of the spectra of the corresponding homoleptic assemblies. This might be a result of a different conformational landscape accessible in the heteroleptic cage (i.e., causing different degrees of twisting between the π‐systems in the backbone of **D2**) or a sign for ground‐state electronic interaction between the ligands. However, the distance between donor **D2** and acceptor **A2** in the X‐ray crystal structure of [Pd_2_ **D2**_2_ **A2**_2_]^4+^ amounts to around 10 Å, too far to expect π‐stacking effects between the chromophores, thus supporting the first reasoning. For both cages, absorptions > 350 nm mostly stem from the donor ligand, allowing for its nearly selective excitation with pump pulses between 375 and 400 nm in the subsequent time‐resolved studies.

The free donor ligands **D1** and **D2** both show a steady‐state emission with a maximum at 500 nm (Figure S45).^[^ ^48^ ^]^ In the homoleptic donor cages [Pd_4_ **D1**_8_]^8+[^ ^54^ ^]^ and [Pd_2_ **D2**_4_]^4+^, the emission is completely quenched, pointing toward the population of dark states involving the Pd(II) nodes. Acceptor ligand **A2** shows an emission maximum at 530 nm. The corresponding homoleptic assembly shows a slightly blueshifted emission which is—contrasting with the homoleptic donor cages—not completely quenched (as previously reported by us^[^ ^49^ ^]^). Within the donor–acceptor cages, however, the emission of both ligands was found to be quenched, which provided a first indication to the occurrence of ligand‐to‐ligand electron transfer as competing deactivation pathway for the electronically excited state.

### Electrochemistry

The cyclic voltammogram of donor–acceptor cage [Pd_2_ **D1**_2_ **A2**_2_]^4+^ in CH_3_CN shows a redox event at E_p/2 _= +0.47 V with peak potentials of E_p1_ = +0.51 V and E_p2_ = +0.42 V, stemming from the oxidation of the phenothiazine donor ligand (Figure 4b). The redox event is anodically shifted compared to the one of the free ligand (E_p/2 _ =  +0.40 V in CH_3_CN) but not as positive as compared to the one of the homoleptic double cage (E_p/2 _ =  +0.52 V in CH_3_CN).^[^ ^33^ ^]^ The anodic shift upon cage formation can probably be traced back to the reduced electron density upon coordination to the metal cations, resulting in an overall positive charge of the cage. The interlocked double cage possesses an even higher positive charge, explaining its more positive half‐wave potential. Furthermore, the cyclic voltammogram of [Pd_2_ **D1**_2_ **A2**_2_]^4+^ displays a redox event at E_p/2 _ =  −1.60 V with peak potentials of E_p1_  =  −1.67 V and E_p2_  =  −1.54 V. The event corresponds to the first reduction of the acceptor **A2** (free ligand **A2**: E_p/2 _ =  −1.54 V in DMSO, Figure S50).

Having the half‐wave potentials of donor and acceptor at hand, we studied the changes in absorbance upon oxidation and reduction of the cage. These spectra will be required for interpreting the transient absorption spectra. Upon application of a potential of + 0.60 V, the steady‐state absorbance of [Pd_2_ **D1**_2_ **A2**_2_]^4+^ between 350 and 450 nm is bleached. Concomitantly, a new feature at 520 nm with a shoulder at higher energies as well as broad absorbance between 600 and 800 nm arises. This absorption pattern is characteristic of the phenothiazine radical cation.^[^ ^32^, ^33^ ^]^ Application of a negative potential (−1.80 V) led to a rise of the absorption between 300 and 400 nm and a broad absorption between 400 and 700 nm. A similar absorption pattern was obtained upon reduction of the free ligand **A2** (Figure S54). The cyclic voltammogram of cage [Pd_2_ **D2**_2_ **A2**_2_]^4+^ shows a redox wave at E_p/2 _ =  +0.34 V, stemming from the oxidation of the triarylamine‐based donor ligand, as well as at E_p/2 _ = −1.49 V, assignable to the reduction of the acceptor ligand **A2**. Oxidation of the donor **D2** is associated with an increase in absorption between 450 and 500 nm as well as the rise of a broad band between 650 and 1000 nm, typical for the formation of the triarylamine radical cation (Figure S53).^[^ ^55^, ^56^, ^57^ ^]^ Overall, only minor shifts of the half‐wave potentials as well as similar spectroscopic features upon oxidation and reduction of the donor–acceptor cages as compared to their free ligands show that the redox‐active moieties remain independently addressable in the electronic ground state of the mixed‐ligand cages.

### Transient Absorption Spectroscopy

Femtosecond pump‐probe transient absorption (TA) spectroscopy was then applied to investigate the photo‐induced excited state dynamics of the free ligands **A2** and **D2**, the corresponding homoleptic assemblies, as well as the donor–acceptor cages. For the UV–vis experiments the compounds were investigated in DMSO solution. As ligand **A2** carries a carbonyl group, giving rise to characteristic vibrational modes,^[^ ^58^, ^59^ ^]^ TA spectra were also recorded in the mid infrared. For the latter method, the compounds must be dissolved in a solvent with sufficient transmission in this spectral range, for which CH_3_CN was chosen. Experimental time traces were analyzed by exponential fitting. The effective time resolution in the UV‐vis and IR was about 100 and 500 fs, respectively.

**Figure 5:**
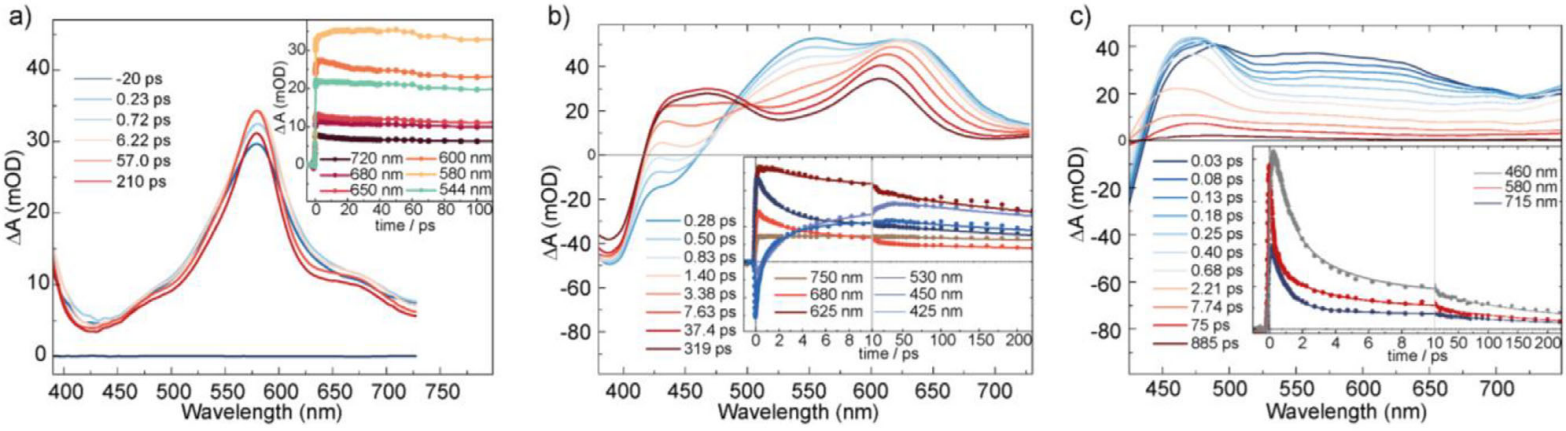
UV–vis transient difference spectra (DMSO, *λ*_ex_ = 375 nm) of a) fluorenone‐based ligand **A2**; b) triarylamine‐based ligand **D2**; and c) homoleptic cage [Pd_2_ **D2**_4_]^4+^; the insets show decay traces at selected wavelengths.

Upon photoexcitation of ligand **A2**, an excited state with an absorption maximum at 570 nm is formed (Figure 5a). This state lives beyond the experimentally accessible time window of 1 ns and minor changes occurring on a 10 ps timescale can probably be explained by intra‐ and/or intermolecular vibrational relaxation processes. As the free ligand exhibits appreciable fluorescence, we assign these transients to the S_1_ state of **A2**. This is supported by the measured emission maximum of **A2** in acetonitrile at 530 nm, which is very similar to the S_1_ fluorescence of the underlying 9‐fluorenone chromophore (maximum at 520 nm) with a lifetime of 15–16 ns in polar solvents.^[^ ^60^ ^]^ The S_1_ fluorescence must contribute a S_1_ → S_0_ stimulated emission (SE) component with negative amplitude to the TA signal. Apparently, this contribution is covered by a larger S_1_ → S*_n_* excited state absorption, which results in an overall positive TA signal at 530 nm. The homoleptic assemblies (mixture of [Pd_3_ **A2**_6_]^6+^ and [Pd_4_ **A2**_8_]^8+^ in CH_3_CN) were investigated in the mid IR probe range (Figure S61). Upon photoexcitation, the ground state vibrational modes at 1447, 1466, 1608, 1620, and 1720 cm^−1^ are instantaneously bleached and a long‐lived state with new absorption bands at 1423, 1479, 1537, and 1596 cm^−1^ appears, which we assign to the S_1_ excited ligands in [Pd_3_ **A2**_6_]^6+^ and [Pd_4_ **A2**_8_]^8+^. That coordination to Pd(II) has only a small effect on the photophysical properties at early time delays is supported by the similar steady‐state emission spectra of free **A2** as compared to its homoleptic assemblies. This observation is furthermore in accordance with our previous observations made for ligand **A1** and its homoleptic assembly [Pd_4_ **A1**_8_]^8+^.^[^ ^33^, ^34^ ^]^

Free donor ligand **D2** exhibits more complex excited state dynamics than the free acceptor **A2** as shown in Figure 5b. UV excitation at 375 nm induces an instantaneous negative absorbance change with a minimum at 400 nm and a shoulder at 440 nm, and broad positive bands with maxima at 550 and 630 nm. The 400 nm minimum is caused by ground state bleaching as it coincides with the steady‐state absorption maximum of **D2** (Figure S41), whereas the 440 nm shoulder is the result of stimulated emission from a locally excited singlet state S_1,L_. The positive bands indicate excited state absorption from S_1,L_ into higher laying singlet states. Spectral evolution and time traces in the inset in Figure 5b show disappearance of the 440 nm stimulated emission and the excited state absorption bands and formation of a new species with absorption maxima at 470 and 610 nm, consistent with an S_1,L_ lifetime of τ_L_ = (2.0 ± 0.2) ps and transformation into a new species with a nanosecond lifetime. As ligand **D2** exhibits reasonable steady‐state fluorescence (Fig. S45) with a maximum at 485 nm, i.e., notably redshifted to the 440 nm stimulated emission of S_1,L_, the newly formed state can be assigned to a singlet, probably with increased charge transfer character, S_1,CT_. It appears that for the transients of S_1,CT_, the stimulated emission component is covered by a stronger excited state absorption resulting in positive Δ*A* values at *λ* > 420 nm.

**Figure 6:**
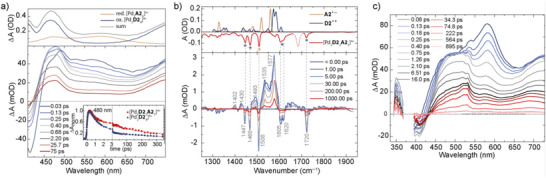
a) Top: UV–vis difference spectra of electrochemically oxidized homoleptic cage [Pd_2_ **D2**_4_]^4+^, reduced [Pd_3_ **A2**_6_]^6+^, and the sum of the spectra (DMSO, 0.1 M TBAPF_6_, WE: platinum gauze, CE: platinum wire, RE: Ag/AgNO_3_, cuvette pathlength: 1.0 mm); bottom: UV–vis transient difference spectra of [Pd_2_ **D2**_2_ **A2**_2_]^4+^ (DMSO, *λ*_ex_  =  375 nm), the inset shows the normalized decay traces at *λ*_probe_  =  480 nm for [Pd_2_ **D2**_4_]^4+^ and of [Pd_2_ **D2**_2_ **A2**_2_]^4+^; b) top: FTIR spectrum of [Pd_2_ **D2**_2_ **A2**_2_]^4+^ (red) and DFT‐computed spectra of ligand radicals **A2^•−^** (orange) and **D2^•+^** (blue); bottom: IR transient difference spectra of [Pd_2_ **D2**_2_ **A2**_2_]^4+^ (CH_3_CN, *λ*_ex_  =  400 nm); c) UV–vis transient difference spectra of [Pd_2_ **D1**_2_ **A2**_2_]^4+^ (CH_3_CN, *λ*_ex_ = 385 nm).

The photoinduced dynamics of the donor ligand dramatically change when it is bound to Pd(II) in the homoleptic cage [Pd_2_ **D2**_4_]^4+^. The spectrum recorded 30 fs after 375 nm excitation of [Pd_2_ **D2**_4_]^4+^ (Figure 5c) indicates some similarities to the S_1,L_ transients of ligand **D2**; however, the absorptions at 550 and 630 nm appear significantly broadened. A band located at 490 nm showing some similarity to the 470 nm feature of the S_1,CT_ state of ligand **D2** might already indicate some intra‐ligand charge transfer contribution. In any case, this initial spectrum, which we attribute to the S_1_ state of donor **D2** in the complex [Pd_2_ **D2**_4_]^4+^, disappears with a time constant of τ_S1 _= (180 ± 40) fs as derived from the decay at 580 nm. This short lifetime is consistent with the observation that the steady‐state emission of [Pd_2_ **D2**_4_]^4+^ is completely quenched (Figure S45). The S_1_ decay leads to a product exhibiting a broad positive difference spectrum with a maximum at 470 nm and onset of a second maximum at > 750 nm. It shows similarities to a spectrum constructed from the difference of the electrochemically oxidized [Pd_2_ **D2**_4_] cage and the [Pd_2_ **D2**_4_]^4+^ ground state spectrum (Figure 6a), suggesting a ligand‐to‐metal charge transfer (LMCT) and transient formation of [Pd(I)Pd(II)**D2**_3_(**D2^•+^**)]^4+^ with one Pd(I) center and a donor radical cation. The decay of this LMCT state, as derived from the decay of the 470 nm band, proceeds on at least two timescales: a dominant contribution with time constant τ_rec1_ = 1.3 ps (75%) and a minor contribution with τ_rec2_ = 200 ps (25%). The LMCT lifetime appears to be significantly longer than the one observed for the homoleptic double cage [Pd_4_ **D1**_8_]^4+^ featuring the other donor alternative.^[^ ^33^, ^34^ ^]^

Concerning the donor–acceptor cage [Pd_2_ **D2**_2_ **A2**_2_]^4+^, its steady‐state absorption spectrum in the near UV region is dominated by the donor ligand whose absorption is about 10 times stronger than the one of the acceptor. Accordingly, excitation of [Pd_2_ **D2**_2_ **A2**_2_]^4+^ at the used laser pump wavelengths 375 and 400 nm produces UV–vis transients, which are very similar to the homoleptic donor cage [Pd_2_ **D2**_4_]^4+^, indicating rapid oxidation of the donor (S_1_ lifetime τ_S1_ = (200 ± 50) fs) and formation of the radical cation **D^•+^** within the cage assembly (Figure 6a). The absorption pattern of **D2^•+^** at 480 nm subsequently decays on timescales of τ_rec1_  =  0.9 ps (relative amplitude 56%), τ_rec2_  =  100 ps (14%), and τ_rec3_  =  500 ps (30%). A comparison of the normalized time traces at 480 and 580 nm with the ones of the homoleptic cage [Pd_2_ **D2**_4_]^4+^ (Figures 6a and S62) reveals ∼50% higher absorption amplitudes for the donor–acceptor cage [Pd_2_ **D2**_2_ **A2**_2_]^4+^ at pump‐probe delays > 2 ps when the singlet state decay is over. This can be explained by a higher yield of **D2^•+^** and/or parallel formation of a radical anion **A2^•−^** as the latter shows a broad, almost unstructured absorption over the entire visible region (Figure 6a). On the basis of the data in the visible region alone, however, it was impossible to clarify whether the electron released by the donor mainly hops to a Pd(II) node to form Pd(I) or to an acceptor ligand to generate **A2^•−^**.

At this point, the IR transient spectra of [Pd_2_ **D2**_2_ **A2**_2_]^4+^ become instrumental (Figure 6b). They clearly show that preferential excitation of ligand **D2** at 400 nm not only bleaches corresponding donor ligand bands (at 1447, 1466, 1605, 1620, and 1720 cm^−1^, marked by blue asterisks in the FTIR spectrum) but also the 1508 cm^−1^ vibration (marked by a beige asterisk), which belongs to the acceptor ligand. Moreover, several positive absorption bands appearing at 1402, 1430, 1493, 1535, and 1577 cm^−1^ agree with the computed IR transitions (B3LYP/6–31g++(d,p), implicit solvent)^[^ ^61^ ^]^ for the radical ions **A2^•−^** and **D2^•+^**, suggesting a ligand‐to‐ligand charge transfer (LLCT) after local excitation of the **D2** chromophore, resulting in the product [Pd_2_(**D2^•+^**)(**D2**)(**A2^•−^**)(**A2**)]^4+^. The two strongest bands at 1535 and 1577 cm^−1^ coincide with IR transitions at 1540 and 1580 cm^−1^ found for the bare fluorenone radical anion.^[^ ^62^ ^]^ Hence, we attribute these features to **A2^•−^** within the charge transfer compound.

Furthermore, the decay of the radical ion bands reports on the lifetime of the charge separated states. Bi‐exponential fitting applied to the 1577 cm^−1^ band results in two time constants of τ_rec2_  =  (70 ± 20) ps and τ_rec3_  =  (1.1 ± 0.4) ns with similar amplitude, agreeing reasonably well with the two slower recombination time constants found in the UV–vis probe region. Assuming that the electron transfer rate depends exponentially on the distance,^[^ ^14^ ^]^ these two components may arise from two different [Pd_2_(**D2^•+^**)(**D2**)(**A2^•−^**)(**A2**)]^4+^ species where the **A2^•−^** and **D2^•+^** ligands are either positioned in *cis* or *trans* configuration, respectively. The prominent recombination process **D2^•+^** + e^−^ → **D2** observed in the UV–vis with a time constant of τ_rec1_  =  0.9 ps is not directly seen in the IR, confirming that the IR excited state absorptions in Figure 6b (in particular at 1535 and 1577 cm^−1^) correspond predominantly to **A2^•−^**. However, this reaction channel manifests itself in the temporal evolution of the two bleached ground state absorption bands at 1508 and 1720 cm^−1^, both exhibiting an additional time constant of τ_VET_ = (23 ± 7) ps accounting for about 50% of the recovery. Fast formation of a **D2^•+^** Pd(I) couple and its recombination within 0.9 ps effectively transforms the pump photon energy into intramolecular vibrational excitation, accompanied by broadening of the ground state IR bands and reduced peak intensities. The excess energy is released to the solvent typically on a 10 ps timescale by vibrational energy transfer (VET),^[^ ^63^ ^]^ consistent with the observed value of τ_VET_.

Concerning the cage with the phenothiazine donor, shortly after 385 nm excitation, the transient UV–vis spectra of [Pd_2_ **D1**_2_ **A2**_2_]^4+^ in CH_3_CN (Figure 6c) show a strong absorption band at 585 nm, which subsequently decays and transforms into a product spectrum with characteristic absorptions at 520 and 700 nm. All these features are well known from our previous study and represent distinct markers of the singlet state of **D1** (585 nm) and its radical cation **D1^•+^** (520 and 700 nm), respectively.^[^ ^33^ ^]^ The transformation **D1**_S1_ → **D1^•+^** proceeds on a timescale of τ_S1_ = (0.25 ± 0.05) ps. The lifetime of **D2^•+^** was determined at a probe wavelength of 510 nm where **D1**_S1_ and **D1^•+^** show an isosbestic point. The analysis reveals three components τ_rec1_ = (1.1 ± 0.2) ps (relative amplitude 55%), τ_rec2_ = (110 ± 10) ps (41%), and τ_rec3_ > 2 ns (4%), which we attribute to the population of the three different photoproducts as follows. Analogous to the interpretation of the transient difference spectra of [Pd_2_ **D2**_2_ **A2**_2_]^4+^, we assign τ_rec1_ to charge recombination of a transiently formed [Pd(I)Pd(II)(**D1^•+^**)(**D1**)(**A2**)_2_]^4+^ species. The two longer lifetimes can be attributed again to charge recombination events of the donor–acceptor CT species [Pd_2_(**D1^•+^**)(**D1**)(**A2^•−^**)(**A2**)]^4+^ with **A2^•−^** and **D1^•+^** ligands being either in *cis* (corresponding to shorter τ_rec2_) or *trans* (longer τ_rec3_) stereoconfiguration (note, however, that the relative amplitudes observed for τ_rec2_ and τ_rec3_ of [Pd_2_ **D2**_2_ **A2**_2_]^4+^ follow an opposite trend, presumably caused by different trends in the formation kinetics of the respective charge separated states).

### Computational Studies

**Figure 7:**
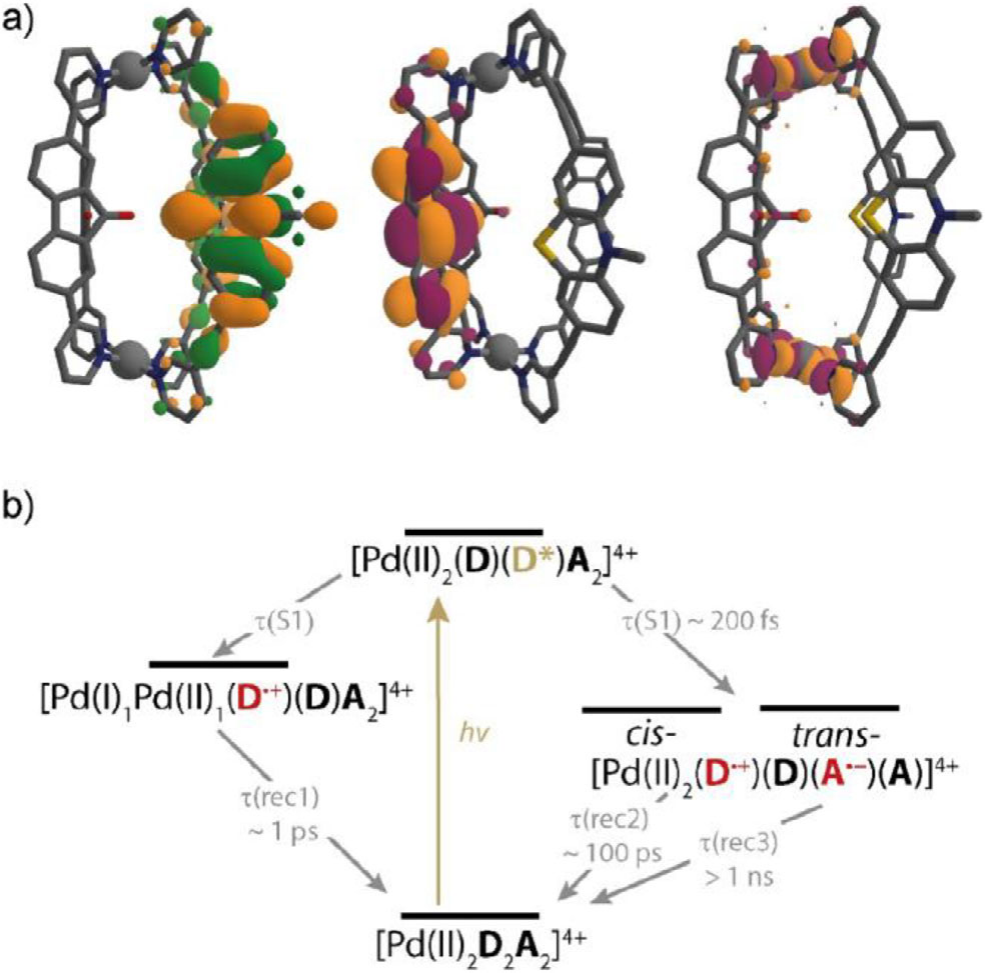
a) Selected molecular orbitals of [Pd_2_ **D1**_2_ **A2**_2_]^4+^: HOMO, LUMO, LUMO + 2 (single point energy calculation B3LYP/Def2‐TZVP); b) schematic representation of photoinduced processes in the investigated [Pd_2_ **D**_2_ **A**_2_]^4+^‐type donor–acceptor cages.

For visualization of the involved molecular orbitals and for approximating their relative energy levels, we modelled the donor–acceptor cages by DFT geometry optimizations (B3LYP/Def2‐SVP), followed by single point energy calculations (B3LYP/Def2‐TZVP). For both donor–acceptor cages, [Pd_2_ **D1**_2_ **A2**_2_]^4+^ and [Pd_2_ **D2**_2_ **A2**_2_]^4+^, the HOMO is located on the donor ligand based on phenothiazine (**D1**) and triarylamine (**D2**), respectively, and the LUMO is located on the fluorenone‐based acceptor ligand **A2** (Figures 7a and S58). The frontier orbital energy levels are furthermore in good accordance with the results obtained by cyclic voltammetry (Table S4). Through a combination of computational, electrochemical, and spectroscopic data, we estimated the driving forces for the respective LMCT and LLCT processes (Table S3). While the calculations are an approximation, since they were carried out in the electronic ground states, the results suggest that the proposed LMCT as well as LLCT processes should be exergonic for both donor–acceptor cages. This supports the conclusions drawn from the transient absorption spectra. Overall, the photoinduced dynamics in the herein introduced class of donor–acceptor cages [Pd_2_ **D**_2_ **A**_2_]^4+^ can be described by the scheme shown in Figure 7b: selective photoexcitation of the donor ligand is followed by ultrafast electron transfer either to one of the acceptor ligands positioned in *cis*‐ or in *trans*‐position to the excited donor or to the Pd(II) node. The LMCT state decays quickly while the LLCT states decay on timescales of hundreds of picoseconds and nanoseconds, respectively.

## Conclusion

Recent progress in establishing robust methods for the non‐statistical, coordination‐driven self‐assembly of multi‐component structures allowed us to obtain a new class of photoredox‐active donor–acceptor cages from simple building blocks in a single step. Therefore, the shape complementary assembly (SCA) approach was employed to guarantee a defined placement of donor and acceptor functionalities within the cages, thus enhancing the modular design and applicability of the herein prepared compounds beyond the previously reported statistical systems that did not allow to derive the required structure–property relationships. Shape complementary donor (**D1**, **D2**) and acceptor (**A2**) ligands were shown to cleanly assemble into two different *cis*‐[Pd_2_(**D1**/**2**)_2_ **A2**_2_]^4+^ cages. The photophysical and electrochemical properties of the donor–acceptor cages indicate that the redox functionalities remain largely independent in the electronic ground state. However, transient absorption spectroscopy in the visible and infrared probe region unambiguously showed that photoexcitation triggers ligand‐to‐ligand electron transfer. This process is in competition with the formation of an LMCT state, owing to the open‐shell nature of the Pd(II) node. Due to the discrete structure of the herein investigated donor–acceptor cages, we were now able to gain a much clearer picture of the photoinduced dynamics in such self‐assembled photoactive systems. Based on the important structure–property relationship data obtained in this study, we are now working on further optimizing our design and extending the scope toward a systematic donor‐acceptor cage library. Access to the non‐statistical assembly of such multicomponent nano‐architectures bears great potential to bring the learned principles and elaborated compound classes into valuable applications concerning the design of novel photovoltaic materials with controlled morphology, guest‐selective sensors and photoredox catalysts for reactions under confinement.

## Supporting Information

The authors have cited additional references within the Supporting Information.^[^ ^64^, ^65^, ^66^, ^67^, ^68^, ^69^, ^70^, ^71^, ^72^, ^73^, ^74^, ^75^, ^76^, ^77^ ^]^ The X‐ray crystallographic coordinates reported in this study have been deposited at the Cambridge Crystallographic Data Centre (CCDC), under deposition numbers 2496389 and 2496390. These data can be obtained free of charge from The Cambridge Crystallographic Data Centre via www.ccdc.cam.ac.uk/data_request/cif.

## Conflict of Interests

The authors declare no conflict of interest.

## Supporting information



Supporting Information

Supporting Information

## Data Availability

The data that support the findings of this study are available from the corresponding author upon reasonable request.
