# Supplementary material for: Non‐Statistical Assembly of Donor–Acceptor Cages for Light‐Induced Charge Separation
Source: Angew Chem Int Ed Engl. 2026 Jan 9;65(7):e22944. doi: 10.1002/anie.202522944 (PMC12887614; doi:10.1002/anie.202522944)
Supplement: Supplementary file 1 — Supporting Information [file ANIE-65-e22944-s001.pdf]

Supporting Information

Non-Statistical Assembly of Donor-Acceptor Cages for Light-Induced Charge Separation

Jacopo Tessarolo,*#[a,b] Laura Neukirch,#[a] Kai Wu,*#[a,c] Jan-Hendrik Borter,[d] Haeri Lee,[e] Dirk Schwarzer,*[d] and Guido H. Clever*[a,f]

[a] Prof. Dr. J. Tessarolo, Dr. L. Neukirch, Prof. Dr. K. Wu, Prof. Dr. G. H. Clever
Department of Chemistry and Chemical Biology, TU Dortmund University
Otto-Hahn Straße 6, 44227, Dortmund, Germany
E-mail: guido.clever@tu-dortmund.de
#These authors have contributed equally

[b] Prof. Dr. J. Tessarolo
Department of Chemistry, Chonnam National University, 77 Yongbong-ro, Buk-gu, Gwangju 61186, Republic of Korea
E-mail: jacopo@jnu.ac.kr

[c] Prof. Dr. K. Wu
Lehn Institute of Functional Materials, Institute of Green Chemistry and Molecular Engineering, Guangdong Basic Research Center of Excellence for Functional Molecular Engineering, MOE Key Laboratory of Bioinorganic and Synthetic Chemistry, School of Chemistry, Sun Yat-Sen University, Guangzhou 510275, China
E-mail: wukai9@mail.sysu.edu.cn

[d] Dr. J.-H. Borter, Prof. Dr. D. Schwarzer
Department of Dynamics at Surfaces, Max-Planck-Institute for Multidisciplinary Sciences
Am Fassberg 11, 37077 Göttingen, Germany
E-mail: dschwar@mpinat.mpg.de

[e] Prof. Dr. H. Lee
Department of Chemistry, Hannam University, Daejeon, Republic of Korea

[f] Prof. Dr. G. H. Clever
Dortmund Center for Advanced Exploration of Dynamics Across Limits Using Spectroscopy (DAEDALUS), TU Dortmund University, Otto-Hahn-Str. 4a, 44227 Dortmund, Germany

Contents

1. General methods 2
2. Synthetic procedures 2
   2.1. Synthesis of ligands 2
   2.2. Self-assembly into coordination cages 6
3. X-ray crystallography 19
4. Photophysical characterization 23
   4.1. Steady state UV-Vis absorption spectroscopy 23
   4.2. Steady state emission spectroscopy 24
   4.3. Steady state IR absorption spectroscopy 27
5. Electrochemistry 29
   5.1. Cyclic voltammetry 29
   5.2. Spectroelectrochemistry 32
6. Computational studies 34
   6.1. Binding angles 34
   6.2. Geometry optimizations and energy calculations 35
   6.3. Calculation of IR absorption spectra 37
7. Transient absorption spectroscopy 38
8. References 41

1. General methods

All chemicals, except otherwise specified, were obtained from commercial sources and used without further purification. Recycling gel permeation chromatography was performed on a JAI LC-9210 II NEXT GPC system or a JAI LaboAce LC-5610 system using chloroform as eluent (HPLC grade). NMR spectroscopic data was measured on the spectrometers Bruker AV 500 Avance NEO, AV 500 Avance III HD, AV 600 Avance III HD, and AV 700 Avance III HD. For ^{1}H and ^{13}C NMR spectra, chemical shifts were calibrated to the solvent lock signal. Chemical shifts δ are given in ppm, coupling constants J in Hz. The following abbreviations are used to describe signal multiplicity for 1H NMR spectra: s: singlet, d: doublet, t: triplet, dd: doublet of doublets; dt: doublet of triplets; m: multiplet, br: broad. All spectra were recorded in standard 5 mm NMR tubes at room temperature. ^{1}H DOSY NMR spectra were recorded with a dstebpgp3s pulse sequence. Mass spectrometry data were measured on Bruker ESI-timsTOF and Bruker compact high-resolution LC mass spectrometers (positive mode). For calibration of the TIMS and TOF devices, Agilent ESI-Low Concentration Tuning Mix was used.

2. Synthetic procedures

2.1. Synthesis of ligands

Ligands **A2** and **D1** were synthesized as previously reported.[49,54]

2.1.1. Synthesis of 3-(7-bromo-9,9-dimethyl-9H-fluoren-2-yl)pyridine **2**

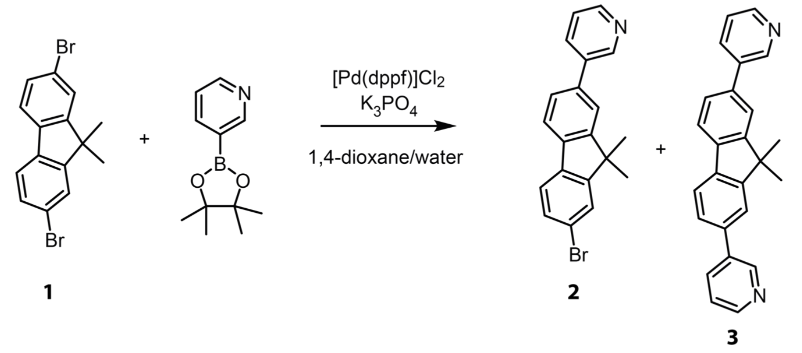

Fig. S1 Synthesis of compound **2**.

A 10 mL aqueous solution of K_3PO_4 (844.1 mg, 3.98 mmol, 2.0 eq.) was added to a 1,4-dioxane (40 mL) solution of 2,7-dibromo-9-fluorenone (701.6 mg, 1.99 mmol, 1.0 eq) and pyridine-3-boronic acid pinacol ester (450 mg, 1.99 mmol, 1.0 eq.). The resulting solution was thoroughly degassed and $[Pd(dppf)_2]Cl_2$ (162.4 mg, 0.2 mmol, 0.1 eq.) was added under nitrogen atmosphere. The mixture was stirred at 90 °C overnight. After cooling down to room temperature, the reaction mixture was extracted with dichloromethane, washed with brine, and dried over $MgSO_4$. The solvent was removed under vacuum, and the crude product was purified through column chromatography (silica) using ethyl acetate:dichloromethane (100:0 → 90:10) as eluent. Monosubstituted product **2** was obtained in a yield of 37% (255.8 mg, 0.73 mmol) along with the disubstituted side product **3** (18%, 129.0 mg, 0.37 mmol).

^1H NMR (500 MHz, Chloroform-*d*) δ 8.91 (s, 1H), 8.61 (s, 1H), 7.98 (d, *J* = 7.8 Hz, 1H), 7.79 (d, *J* = 7.7 Hz, 1H), 7.63 – 7.55 (m, 4H), 7.49 (dd, *J* = 8.0, 1.8 Hz, 1H), 7.43 (br, 1H), 1.53 (s, 6H).

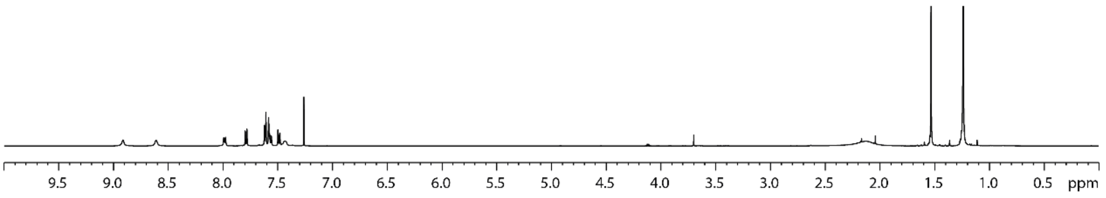

Fig. S2 ^1H NMR of compound **2** (500 MHz, 298 K, $CDCl_3$).

2.1.2. Synthesis of ligand **D2**

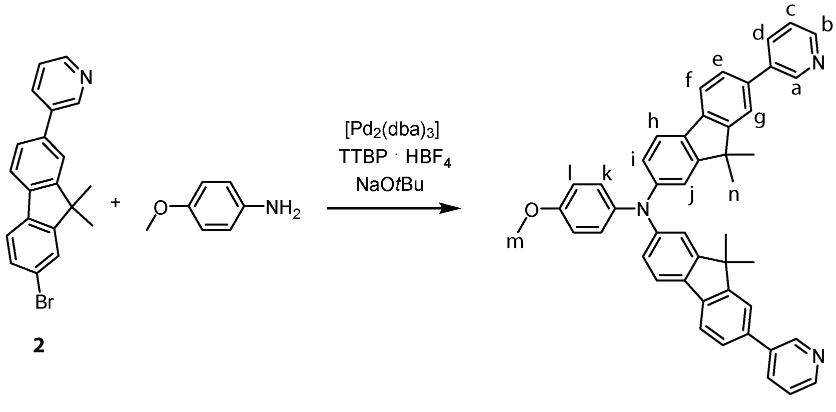

Fig. S3 Synthesis of ligand **D2**.

p-Anisidine (25.49 mg, 207 µmol, 1.0 eq.), compound **2** (163.53 mg, 467 µmol, 2.3 eq.), sodium *tert*-butoxide (58.83 mg, 612 µmol, 3.0 eq.), and tri-*tert*-butylphosphonium tetrafluoroborate (TTBP·HBF_4) (11.93 mg, 41 µmol, 0.2 eq.) were suspended in 25 mL of dry toluene. The reaction mixture was thoroughly degassed and then stirred at 90 °C over night. After cooling down to room temperature, the reaction mixture was extracted with dichloromethane, washed with brine, and dried over $MgSO_4$. The crude reaction product was then purified by column chromatography (silica) using pentane:ethyl acetate (50:50 → 0:100) as eluent. Further purification was carried out by gel permeation chromatography (GPC) using chloroform as eluent, to afford 56.5 mg (85 µmol) of pure **D2** with a yield of 41 % as brown solid.

^{1}H NMR (700 MHz, CD_3CN) δ 8.93 (s, 2H, a), 8.55 (d, *J* = 4.1, 2H, b), 8.05 (ddd, *J* = 7.9, 2.4, 1.6 Hz, 2H, d), 7.79 (m, 4H, f,g), 7.69 (d, *J* = 8.2 Hz, 2H, h), 7.64 (dd, *J* = 7.8, 1.8 Hz, 2H, e), 7.44 (dd, *J* = 7.9, 4.8, 2H, c), 7.27 (d, *J* = 2.1 Hz, 2H, j), 7.16 (dd, *J* = 5.8, 2.1, 2H, l), 7.00 – 6.93 (m, 4H, k,i), 3.81 (s, 2H, m), 1.45 (s, 6H, n).

^{13}C NMR (176 MHz, CD_3CN) δ 156.49, 155.52, 149.26, 149.14, 148.90, 141.42, 139.99, 137.57, 136.80, 135.05, 133.40, 128.58, 127.16, 124.65, 122.75, 122.39, 122.02, 120.92, 117.99, 115.92, 56.12, 47.81, 27.19.

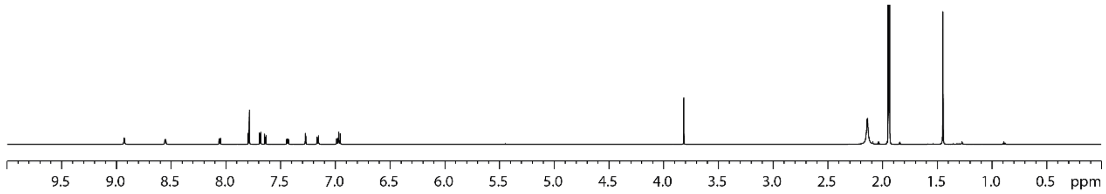

Fig. S4 Full ^{1}H NMR of ligand **D2** (700 MHz, 298 K, CD_3CN).

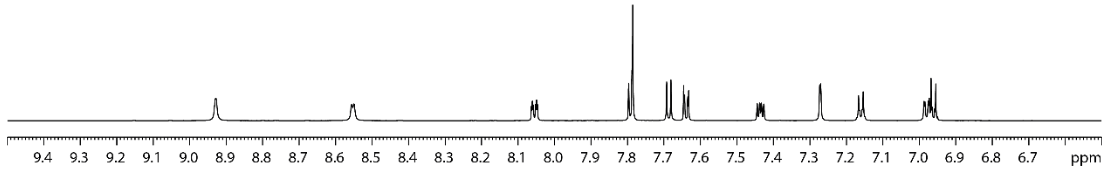

Fig. S5 Partial ^{1}H NMR of ligand **D2** (700 MHz, 298 K, CD_3CN).

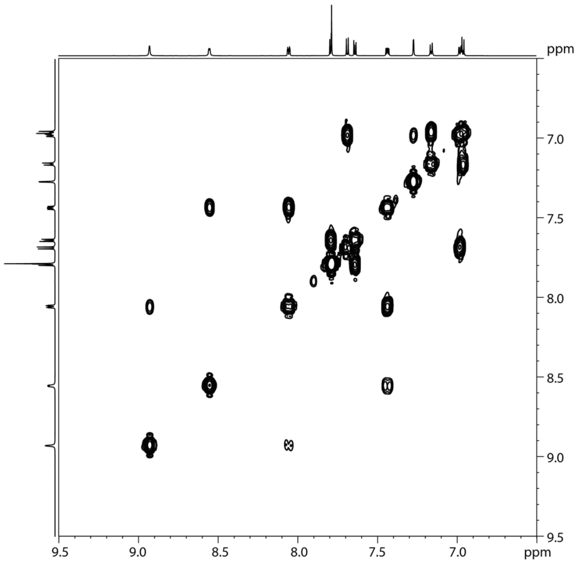

Fig. S6 Partial $^{1}H,^{1}H$-COSY NMR of ligand **D2** (700 MHz, 298 K, CD_3CN).

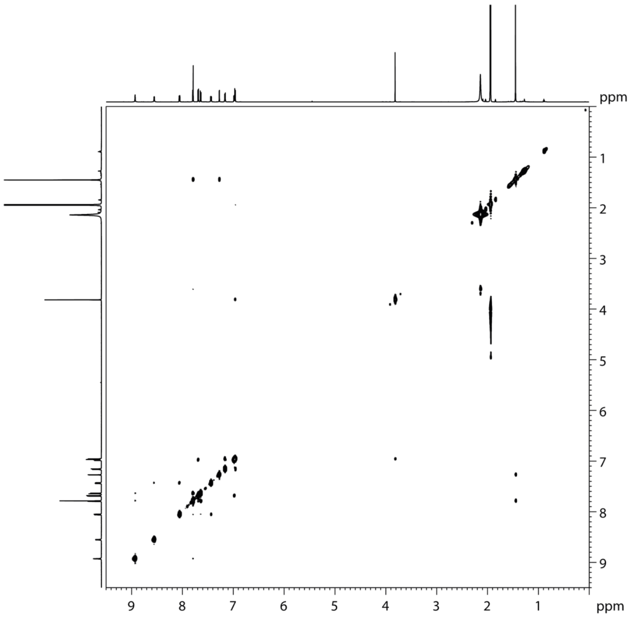

Fig. S7 Full $^{1}H,^{1}H$-NOESY NMR of ligand **D2** (700 MHz, 298 K, CD_3CN).

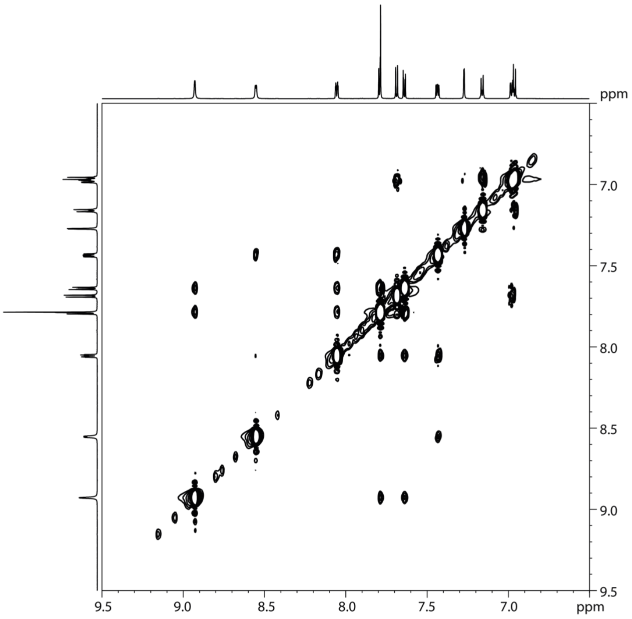

Fig. S8 Partial ^{1}H,^{1}H-NOESY NMR of ligand **D2** (700 MHz, 298 K, CD_3CN).

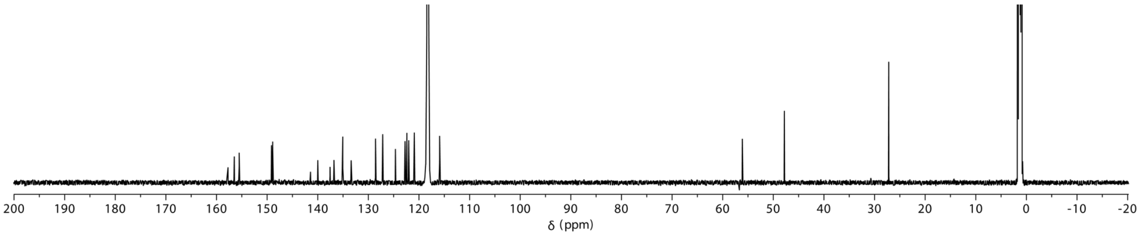

Fig. S9 ^{13}C NMR of ligand **D2** (176 MHz, 298 K, CD_3CN).

2.2. Self-assembly into coordination cages

The self-assembly of ligand **A2** was carried out as reported beforehand.[49]

2.2.1. Synthesis of $[Pd_2\mathbf{D1}_4]^{4+}$

Solutions of $[Pd(CH_3CN)_4](BF_4)_2$ (500 µL, 2.8 mM, 1.4 µmol, 1 eq) and ligand **D1** (500 µL, 2.8 mM, 1.4 µmol, 2 eq) in DMSO-d_6 were combined. The reaction mixture was heated at 70 °C for 1 h. For characterization in CD_3CN, the solvent was then removed via lyophilization and the sample redissolved in CD_3CN. Self-assembly in CD_3CN would lead to interlocked double cage $[Pd_4\mathbf{D1}_8]^{8+}$.[54]

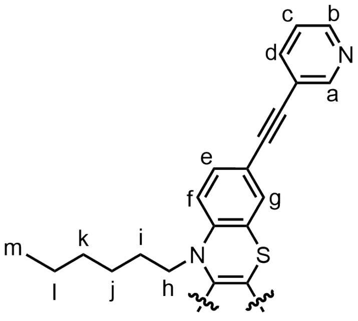

^{1}H NMR (500 MHz, CD_3CN) δ 9.19 (d, *J* = 1.8 Hz, 1H, a), 8.93 (dd, *J* = 6.0, 1.4 Hz, 1H, b), 8.01 (dt, *J* = 8.0, 1.5 Hz, 1H, d), 7.57 (dd, *J* = 7.9, 5.9 Hz, 1H, c), 7.45 – 7.37 (m, 2H, g,e), 6.99 (d, *J* = 8.6 Hz, 1H, f), 3.90 (t, *J* = 7.0 Hz, 1H, h), 1.76 – 1.69 (m, 1H, i), 1.42 – 1.38 (m, 1H, j), 1.29 – 1.23 (m, 2H, k,l), 0.82 (t, *J* = 7.0 Hz, 1.5H, m).

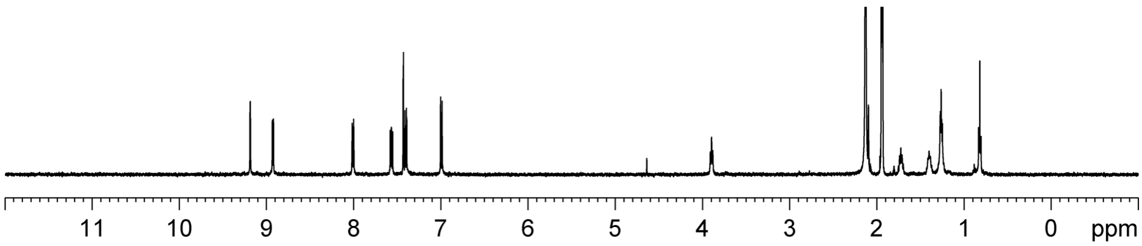

Fig. S10 Full ^{1}H NMR of $[Pd_2\mathbf{D1}_4]^{4+}$ (500 MHz, 298 K, CD_3CN).

2.2.2. Synthesis of $[Pd_2\mathbf{D2}_4]^{4+}$

Solutions of $[Pd(CH_3CN)_4](BF_4)_2$ (42 µL from a 25 mM stock solution, 0.7 µmol, 1 eq) and ligand **D2** (500 µL, 2.8 mM, 1.4 µmol, 2 eq) using DMSO-*d*$_6$ or CD_3CN as solvents, were combined. The reaction mixture was heated at 70 °C for 2 h in both solvent cases. It is worth mentioning that the sample prepared in CD_3CN lead to precipitation after ~48h, still leaving enough time for an NMR characterization.

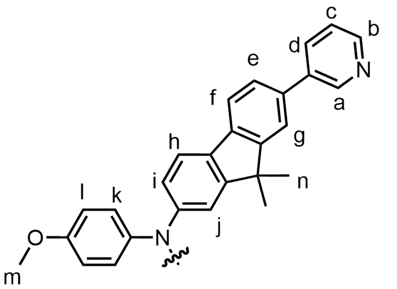

^{1}H NMR (500 MHz, DMSO-*d*$_6$) δ 9.68 (s, 2H, a), 9.39 (d, *J* = 5.7 Hz, 2H, b), 8.42 (d, *J* = 8.0 Hz, 2H, d), 7.91 – 7.81 (m, 6H, c, f, g), 7.77 (d, *J* = 8.2 Hz, 2H, h), 7.63 (d, *J* = 8.0 Hz,

2H, e), 7.22 (s, 2H, j), 7.11 (d, *J* = 8.8 Hz, 2H, k), 6.97 (d, *J* = 8.8 Hz, 2H, l), 6.90 (d, *J* = 8.3 Hz, 2H, i), 3.76 (s, 3H, m), 1.40 (s, 12H, n).

^{1}H NMR (500 MHz, CD_3CN) δ 9.55 (s, 2H, a), 9.00 (d, *J* = 5.8 Hz, 2H, b), 8.27 (d, *J* = 8.1 Hz, 2H, d), 7.82 – 7.74 (m, 4H, g, f), 7.82 – 7.75 (m, 4H, h, c), 7.59 (d, *J* = 7.8 Hz, 2H, e), 7.22 (s, 2H, j), 7.09 (d, *J* = 8.6 Hz, 2H, l), 6.99 – 6.90 (m, 4H, i, k), 3.79 (s, 3H, m), 1.39 (s, 12H, n).

^{13}C NMR (176 MHz, DMSO-d_6) δ 171.34, 156.27, 155.04, 154.21, 149.17, 148.29, 147.73, 139.77, 139.44, 138.65, 132.37, 131.39, 127.50, 127.18, 126.52, 121.57, 121.32, 121.12, 119.99, 117.94, 116.27, 115.08, 55.14, 46.63, 26.61.

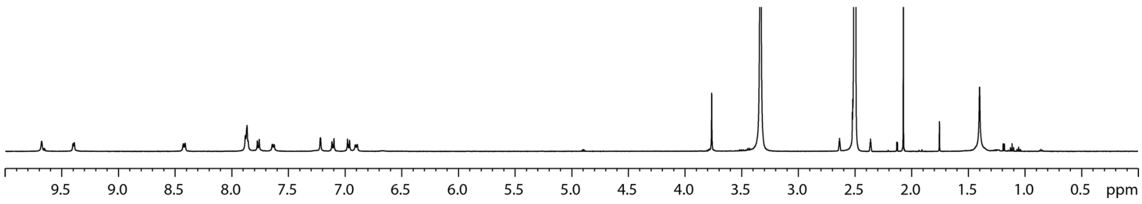

Fig. S11 Full ^{1}H NMR of $[Pd_2\mathbf{D2}_4]^{4+}$ (500 MHz, 298 K, DMSO-d_6).

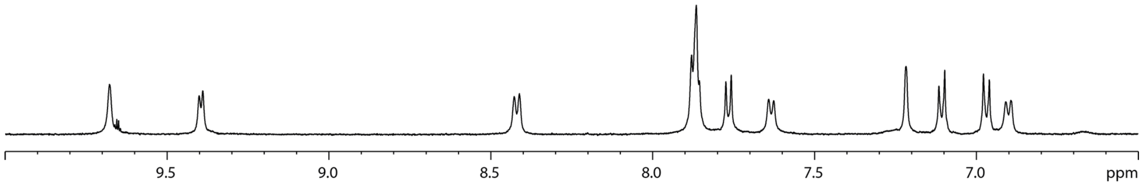

Fig. S12 Partial ^{1}H NMR of $[Pd_2\mathbf{D2}_4]^{4+}$ (500 MHz, 298 K, DMSO-d_6).

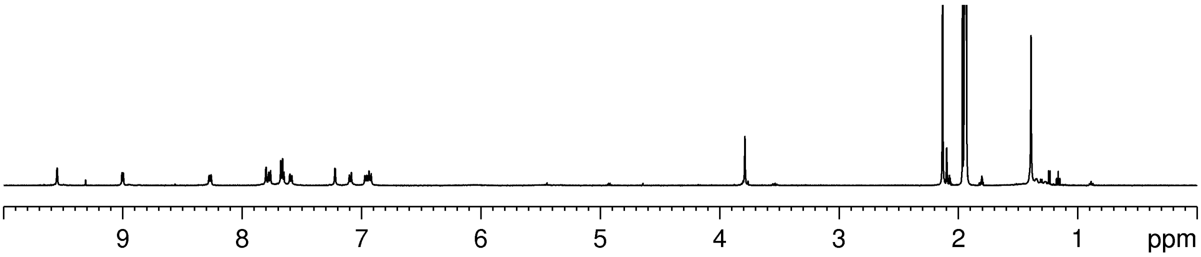

Fig. S13 Full ^{1}H NMR of $[Pd_2\mathbf{D2}_4]^{4+}$ (500 MHz, 298 K, CD_3CN).

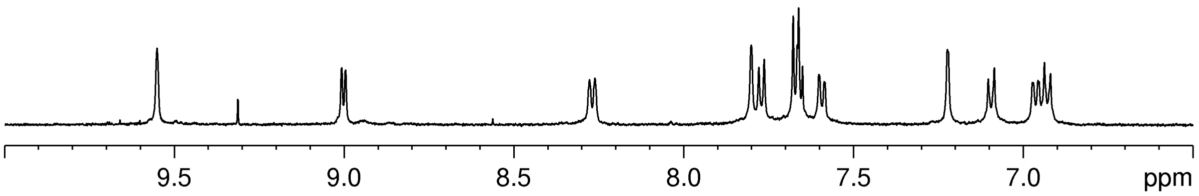

Fig. S14 Partial ^{1}H NMR of $[Pd_2\mathbf{D2}_4]^{4+}$ (500 MHz, 298 K, CD_3CN).

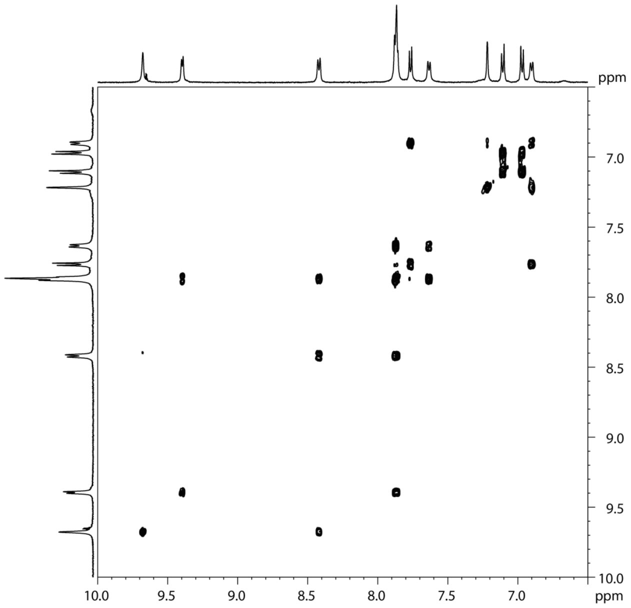

Fig. S15 Partial ^{1}H,^{1}H-COSY NMR of $[Pd_2\mathbf{D2}_4]^{4+}$ (500 MHz, 298 K, DMSO-d_6).

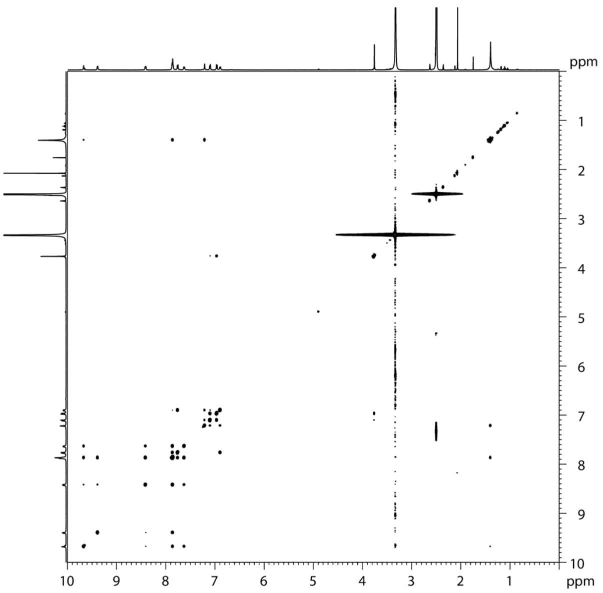

Fig. S16 Full ^{1}H,^{1}H-NOESY NMR of $[Pd_2\mathbf{D2}_4]^{4+}$ (500 MHz, 298 K, DMSO-d_6).

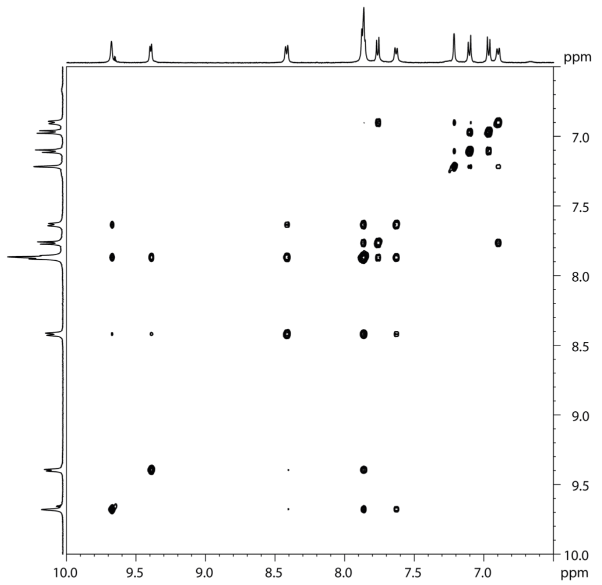

Fig. S17 Partial $^{1}H,^{1}H$-NOESY NMR of $[Pd_2\mathbf{D2}_4]^{4+}$ (500 MHz, 298 K, DMSO-d_6).

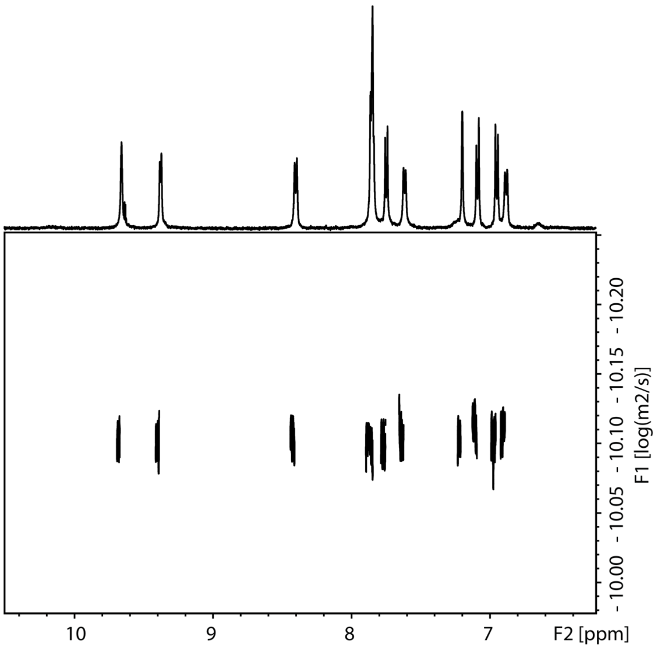

Fig. S18 ^{1}H-DOSY NMR of $[Pd_2\mathbf{D2}_4]^{4+}$, r_H = 12.57 Å (500 MHz, 298 K, DMSO-d_6).

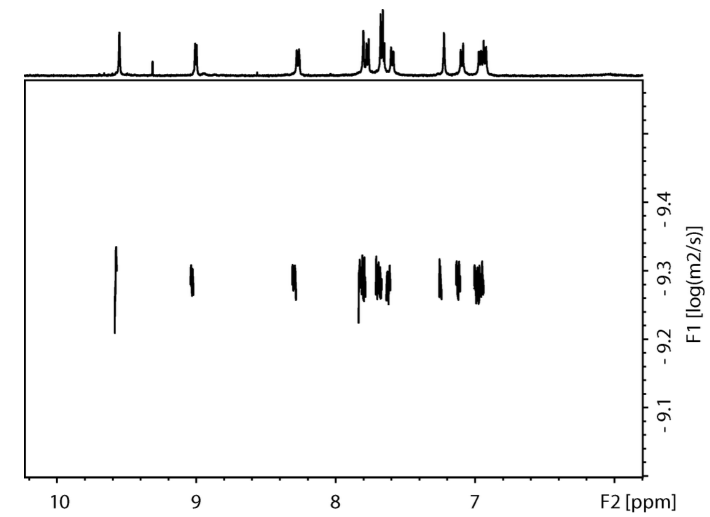

Fig. S19 ^{1}H-DOSY NMR of $[Pd_2\mathbf{D2}_4]^{4+}$, r_H = 10.77 Å (500 MHz, 298 K, CD_3CN).

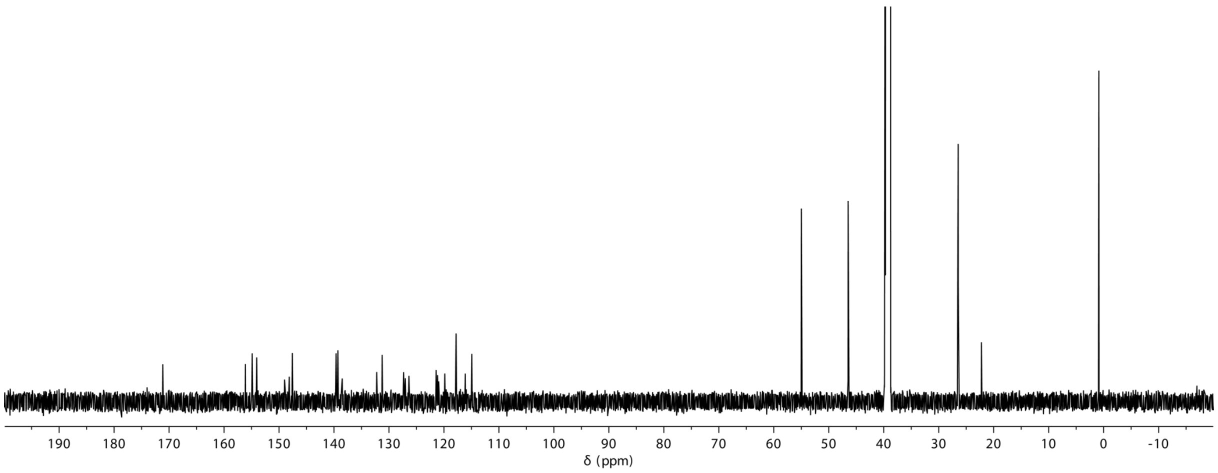

Fig. S20 ^{13}C NMR of $[Pd_2\mathbf{D2}_4]^{4+}$ (176 MHz, 298 K, DMSO-d_6).

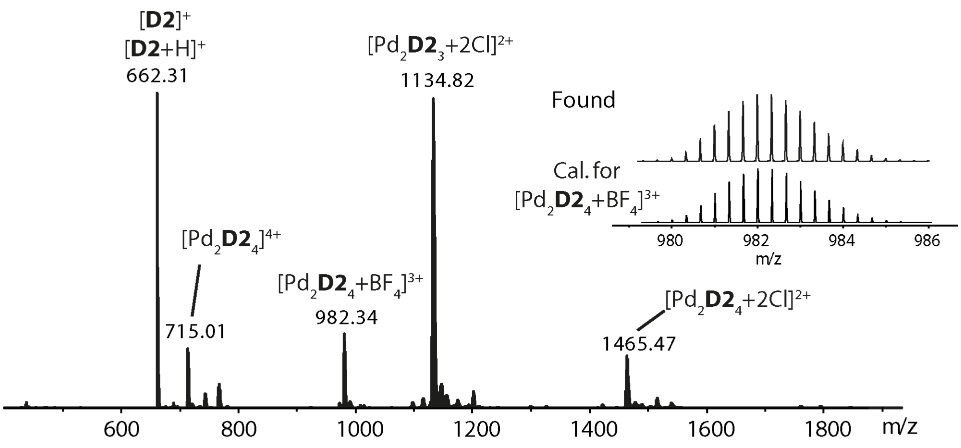

Fig. S21 ESI mass spectrum of $[Pd_2\mathbf{D2}_4]^{4+}$.

2.2.3. Synthesis of $[Pd_2\mathbf{D1}_2\mathbf{A2}_2]^{4+}$

To a solution of **D1** (2 mM, 0.7 µmol) in 350 µL CD_3CN and a suspension of **A** (0.34 mg, 0.7 µmol) in 103 µL CD_3CN was added a stock solution of $[Pd(CH_3CN)_4](BF_4)_2$ (47 µL, 15 mM/CD_3CN, 0.7 µmol). The mixture was heated at 80 °C for 8 h to give a 0.7 mM cage solution.

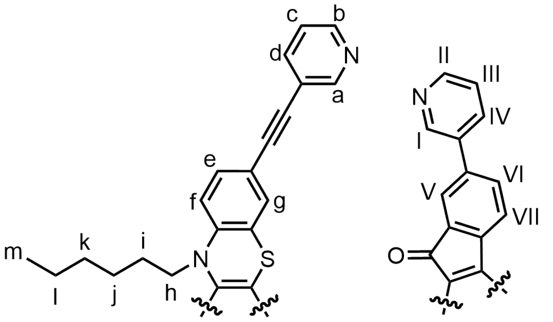

^1H NMR (500 MHz, 298 K, CD_3CN) δ 9.78 (d, *J* = 2.1 Hz, 2H, I), 9.52 (d, *J* = 1.8 Hz, 2H, a), 9.08 (d, *J* = 5.7 Hz, 2H, b), 8.97 (d, *J* = 5.6 Hz, 2H, II), 8.21 (d, *J* = 8.0 Hz, 2H, IV), 8.16 (d, *J* = 1.7 Hz, 2H, V), 7.95 (dt, *J* = 8.1, 1.7 Hz, 2H, d), 7.82 – 7.75 (m, 4H, VII,VI), 7.66 – 7.60 (m, 6H, III,g,c), 7.35 (dd, *J* = 8.4, 2.0 Hz, 2H, e), 6.99 (d, *J* = 8.5 Hz, 2H, f), 3.91 (t, *J* = 7.0 Hz, 2H, h), 1.73 (t, *J* = 7.4 Hz, 2H, i), 1.40 (d, *J* = 7.9 Hz, 2H, j), 1.26 – 1.21 (m, 4H, k,l), 0.84 – 0.78 (m, 3H, m).

^{13}C NMR (151 MHz, 298 K, CD_3CN) δ 192.72, 154.75, 150.82, 149.98, 149.36, 146.71, 145.35, 142.00, 140.56, 139.34, 137.22, 132.55, 131.77, 128.44, 128.40, 125.06, 124.77, 123.37, 123.26, 96.29, 85.30, 48.28, 31.98, 27.10, 26.86, 23.24, 14.21.

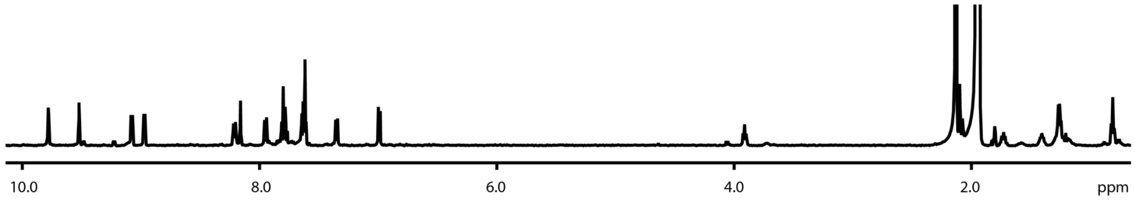

Fig. S22 ^1H NMR of $[Pd_2\mathbf{D1}_2\mathbf{A2}_2]^{4+}$ (500 MHz, 298 K, CD_3CN).

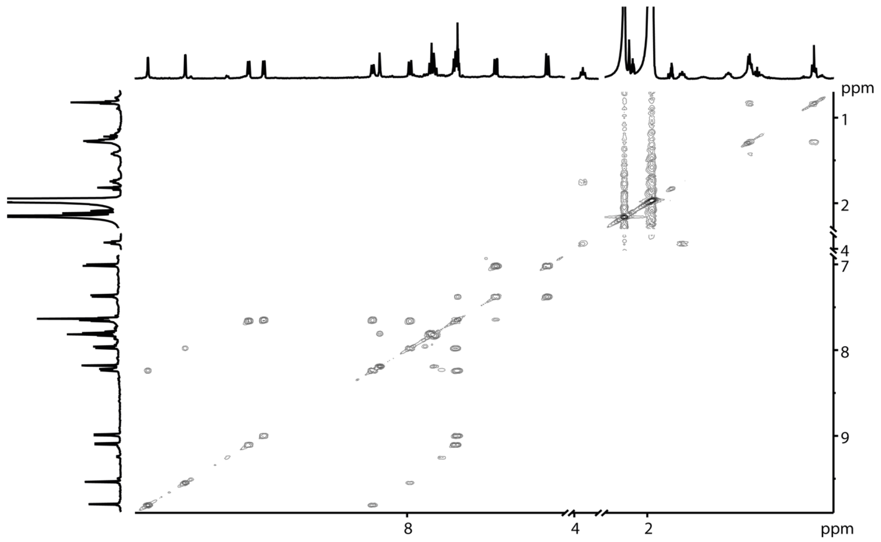

Fig. S23 ^{1}H-^{1}H COSY NMR of $[Pd_2\mathbf{D1}_2\mathbf{A2}_2]^{4+}$ (500 MHz, 298 K, CD_3CN).

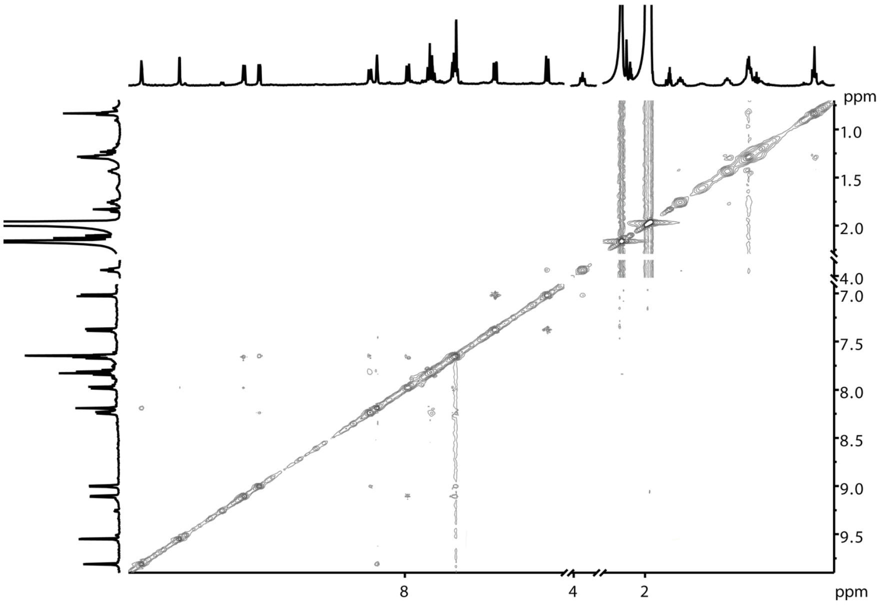

Fig. S24 ^{1}H-^{1}H NOESY NMR of $[Pd_2\mathbf{D1}_2\mathbf{A2}_2]^{4+}$ (500 MHz, 298 K, CD_3CN).

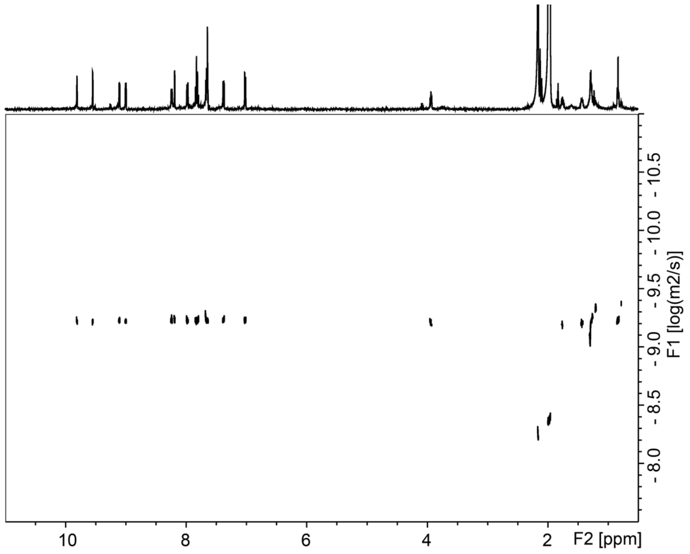

Fig. S25 ^{1}H DOSY NMR of $[Pd_2\mathbf{D1}_2\mathbf{A2}_2]^{4+}$, r_H = 10.9 Å (500 MHz, 298 K, CD_3CN).

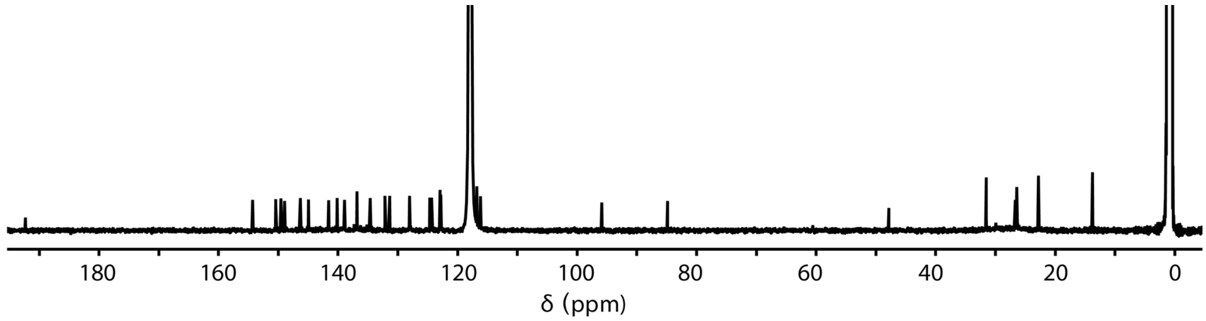

Fig. S26 ^{13}C NMR of $[Pd_2\mathbf{D1}_2\mathbf{A2}_2]^{4+}$ (151 MHz, 298 K, CD_3CN).

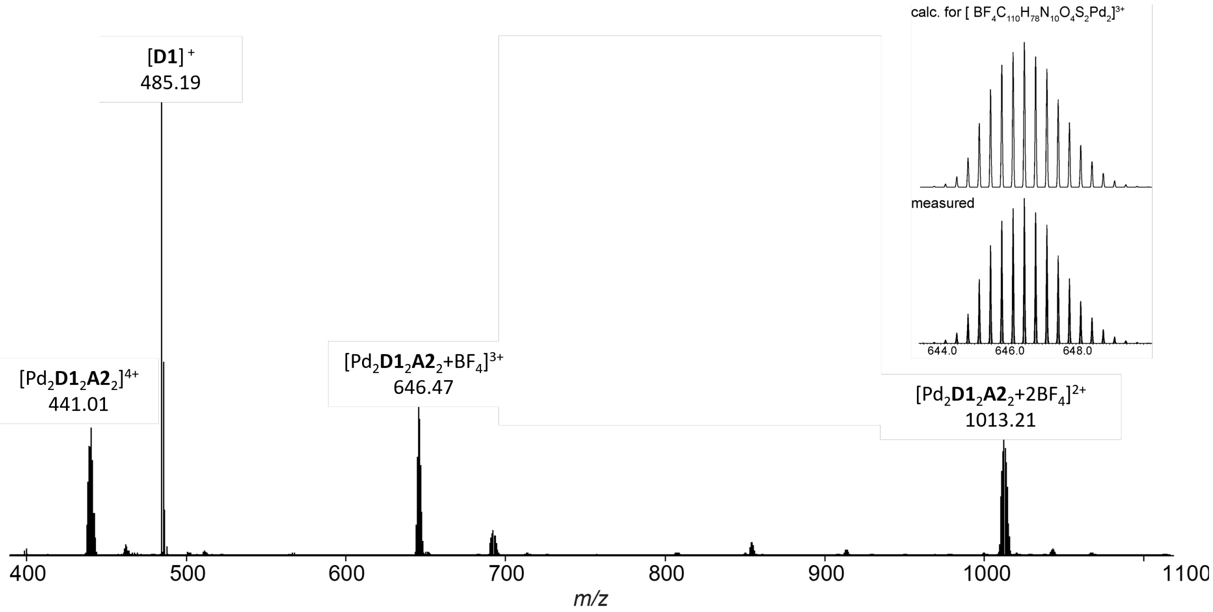

Fig. S27 ESI mass spectrum of $[Pd_2\mathbf{D1}_2\mathbf{A2}_2]^{4+}$.

2.2.4. Synthesis of $[Pd_2\mathbf{D2}_2\mathbf{A2}_2]^{4+}$

Solutions/suspensions of $[Pd(CH_3CN)_4](BF_4)_2$ (42 µL from a 25 mM stock solution, 0.7 µmol, 1 eq), ligand **D2** (250 µL from a 25 mM stock solution, 0.7 µmol, 1 eq), and ligand **A2** (250 µL from a 25 mM stock solution, 0.7 µmol, 1 eq) were combined in either in DMSO-d_6 or CD_3CN. The reaction mixture was heated at 70 °C for 30 min in DMSO-d_6 or overnight in CD_3CN.

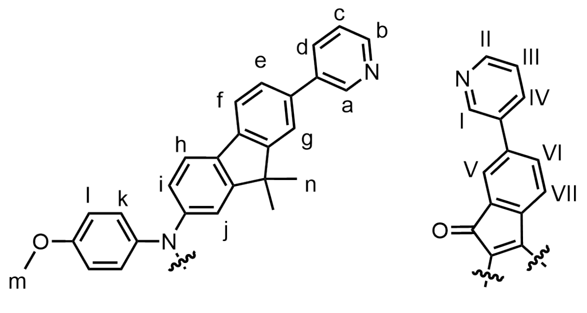

^{1}H NMR (700 MHz, CD_3CN) δ 9.63 (d, *J* = 2.2 Hz, 2H, I), 9.37 (d, *J* = 2.0 Hz, 2H, a), 9.24 (dd, *J* = 5.8, 1.3 Hz, 2H, b), 9.11 (dd, *J* = 5.8, 1.3 Hz, 2H, II), 8.26 – 8.18 (m, 4H, d,IV), 7.91 (s, 2H, V), 7.83 – 7.71 (m, 10H, VI,VII,g,f,c), 7.67 (dd, *J* = 8.0, 5.8 Hz, 2H, III), 7.64 (d, *J* = 8.1 Hz, 2H, h), 7.49 – 7.46 (dd, *J* = 10.0, 2.0 Hz, 4H, e,j), 7.18 – 7.12 (m, 2H, k), 7.00 – 6.94 (m, 2H, l), 6.80 (dd, *J* = 8.0, 2.2 Hz, 2H, i), 3.81 (s, 2H, m), 1.45 (s, 3H, n), 1.44 (s, 3H, n').

^{1}H NMR (500 MHz, DMSO-d_6) δ 10.17 (s, 2H, I), 9.55 (d, J = 5.7 Hz, 2H, b), 9.25 – 9.18 (m, 4H, II, a), 8.49 – 8.42 (m, 4H, IV, d), 8.08 – 7.95 (m, 8H, V, c, VI, VII), 7.85 – 7.70 (m, 8H, f, III, g, h), 7.59 (d, J = 7.9 Hz, 2H, e), 7.33 (d, J = 2.2 Hz, 2H, j), 7.14 (d, J = 8.9 Hz, 2H, k), 6.99 (d, J = 9.1 Hz, 2H, l), 6.76 (dd, J = 8.1, 2.1 Hz, 2H, i), 3.77 (s, 3H, m), 1.40 (s, 3H, n), 1.30 (s, 3H, n').

^{13}C NMR (176 MHz, CD_3CN) δ 193.27, 158.32, 156.09, 155.48, 151.13, 150.29, 149.34, 149.27, 148.98, 145.33, 141.58, 141.44, 140.59, 140.52, 139.86, 139.69, 137.57, 137.01, 135.71, 133.67, 132.68, 129.46, 128.59, 128.35, 127.94, 123.48, 122.52, 122.35, 122.21, 121.20, 116.93, 116.10, 56.15, 47.73, 27.26, 27.24.

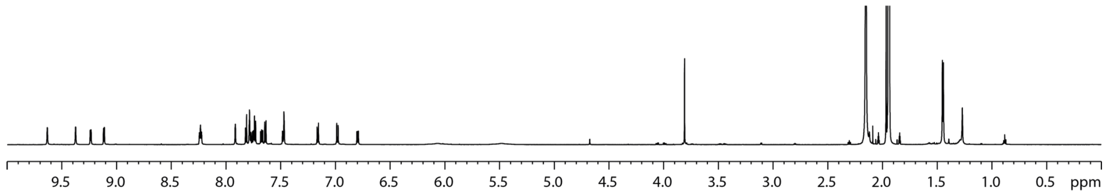

Fig. S28 ^{1}H NMR of $[Pd_2\mathbf{D2}_2\mathbf{A2}_2]^{4+}$ (500 MHz, 298 K, CD_3CN).

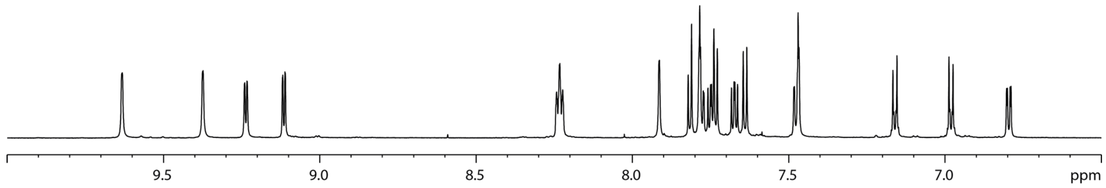

Fig. S29 Partial ^{1}H NMR of $[Pd_2\mathbf{D2}_2\mathbf{A2}_2]^{4+}$ (500 MHz, 298 K, CD_3CN).

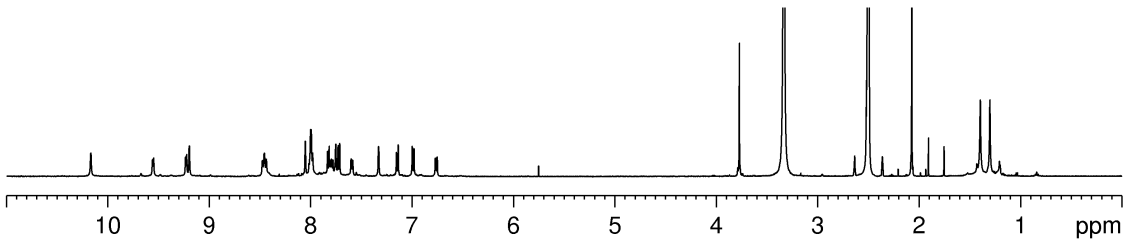

Fig. S30 ^{1}H NMR of $[Pd_2\mathbf{D2}_2\mathbf{A2}_2]^{4+}$ (500 MHz, 298 K, DMSO-d_6).

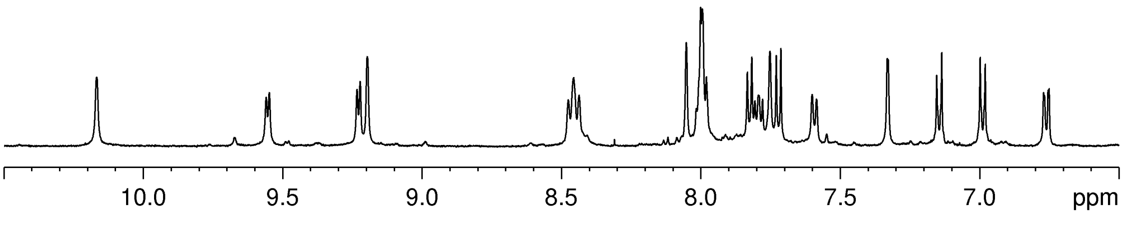

Fig. S31 Partial ^{1}H NMR of $[Pd_2\mathbf{D2}_2\mathbf{A2}_2]^{4+}$ (500 MHz, 298 K, DMSO-d_6).

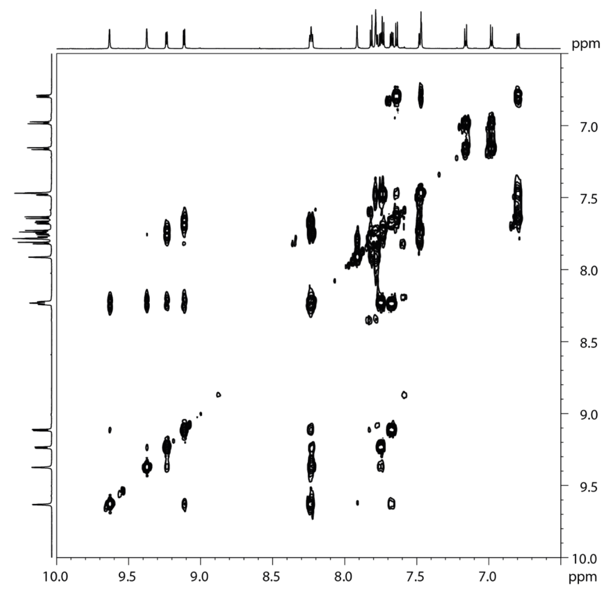

Fig. S32 Partial ^{1}H-^{1}H COSY NMR of $[Pd_2\mathbf{D2}_2\mathbf{A2}_2]^{4+}$ (500 MHz, 298 K, CD_3CN).

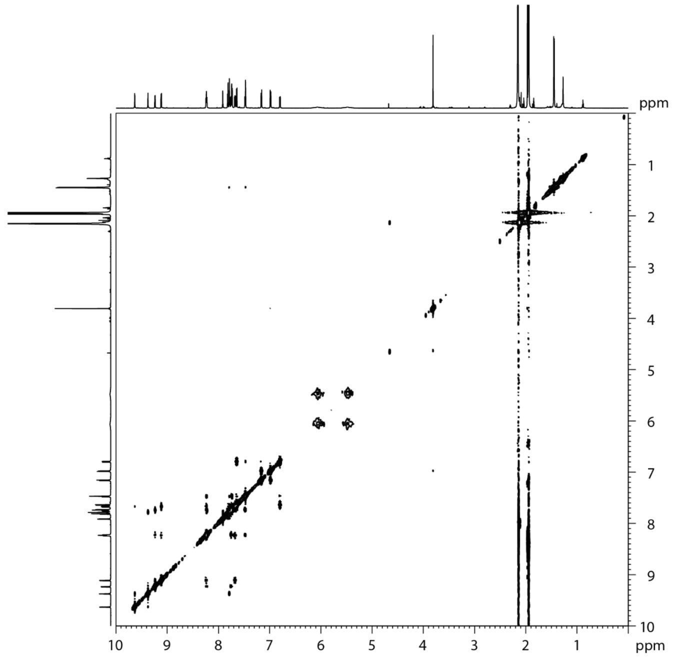

Fig. S33 Full ^{1}H-^{1}H NOESY NMR of $[Pd_2\mathbf{D2}_2\mathbf{A2}_2]^{4+}$ (500 MHz, 298 K, CD_3CN).

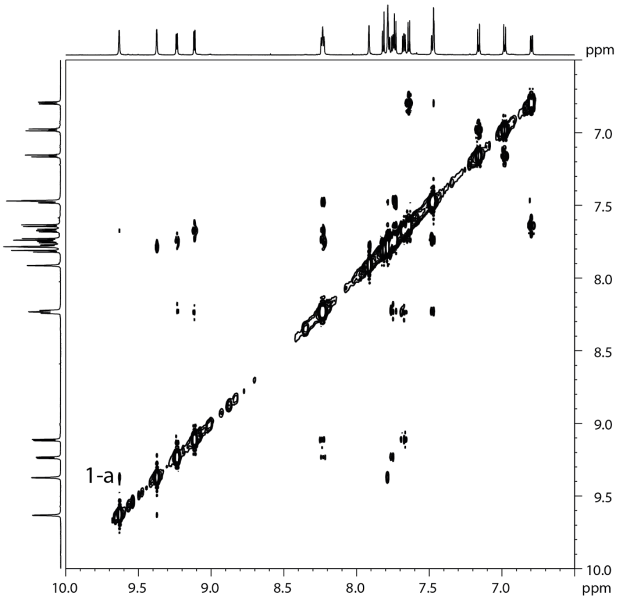

Fig. S34 Partial ^{1}H-^{1}H NOESY NMR of $[Pd_2\mathbf{D2}_2\mathbf{A2}_2]^{4+}$ (500 MHz, 298 K, CD_3CN).

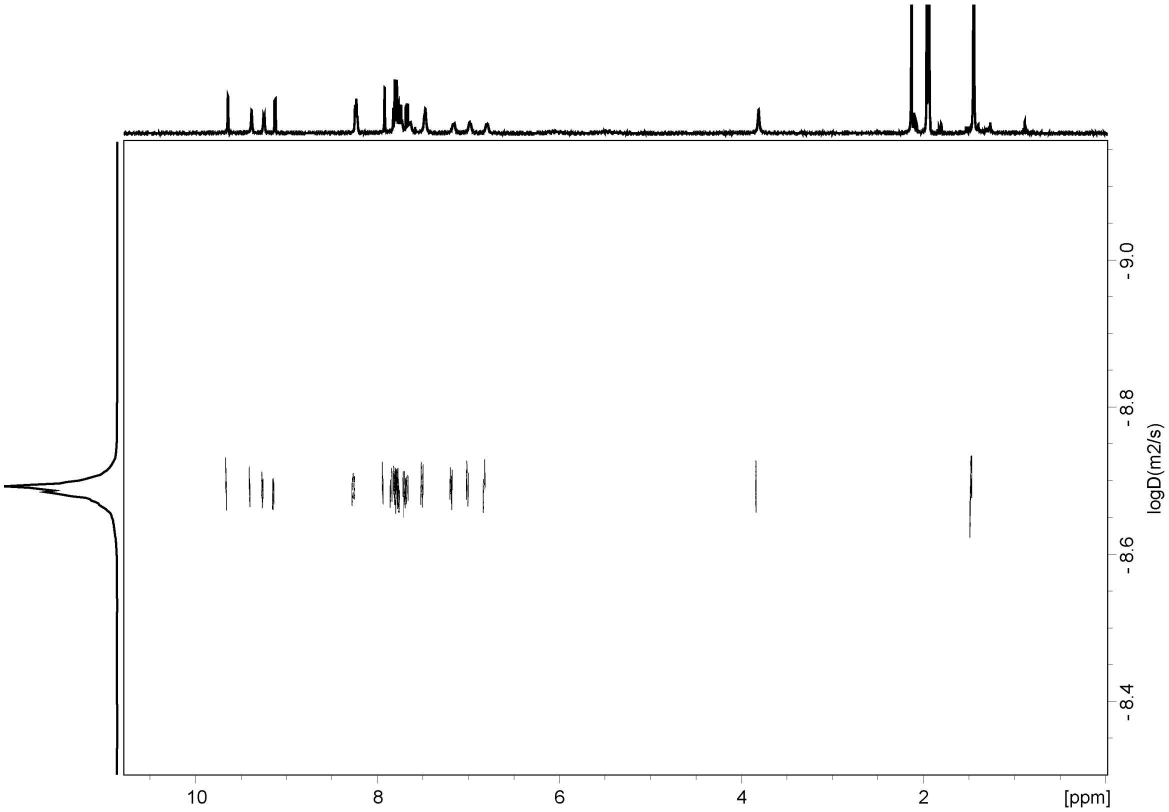

Fig. S35 ^{1}H DOSY NMR of $[Pd_2\mathbf{D2}_2\mathbf{A2}_2]^{4+}$, r_H = 10.29 Å (500 MHz, 298 K, CD_3CN).

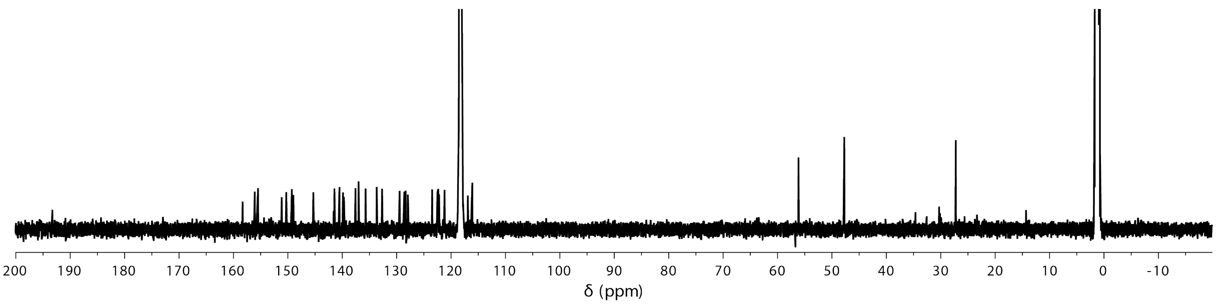

Fig. S36 ^{13}C NMR of $[Pd_2\mathbf{D2}_2\mathbf{A2}_2]^{4+}$ (176 MHz, 298 K, CD_3CN).

3. X-ray crystallography

General Methods

Crystals of supramolecular assemblies were extremely sensitive to loss of organic solvent. Single crystals for X-ray structural analyses of $[Pd_2\mathbf{D2}_4]^{4+}$ and $[Pd_2\mathbf{D2}_2\mathbf{A2}_2]^{4+}$, were mounted at room temperature in NVH oil, then rapidly frozen in liquid N_2. The supramolecular assemblies required synchrotron radiation in order to be successfully determined by single crystal X-ray crystallography. Diffraction data was collected at macromolecular synchrotron beamline P11, PETRA III, DESY.[64] Data integration and reduction were undertaken using XDS.[65] Despite using synchrotron radiation, the analysis was significantly hampered by the limited scattering power due to very small rod- or needle-shaped crystals and the targeted resolution of 0.84 Å for both of $[Pd_2\mathbf{D2}_4]^{4+}$ and $[Pd_2\mathbf{D2}_2\mathbf{A2}_2]^{4+}$ could not be reached in all cases. The resolution was cut off at 1.3083 Å for $[Pd_2\mathbf{D2}_4]^{4+}$, 1.3001 Å for $[Pd_2\mathbf{D2}_2\mathbf{A2}_2]^{4+}$. The structures were solved by intrinsic phasing/direct methods using SHELXT[66] and refined with SHELXL (2019/2)[67] for full-matrix least-squares routines on F^2 and ShelXle[68] as a graphical user interface. The SQUEEZE[69] method provided by the program PLATON[70] was used to improve the contrast of the electron density map the structure. Modelling disorder of flexible ligand, disorder of a full cage over special position as well as counterion and solvent flexibility required carefully adapted macromolecular refinement protocols such as employing geometrical restraint dictionaries, local structural similarity restraints and restraints for anisotropic displacement parameters (ADPs). Analyzing morphologies and detailed geometries greatly enhanced the in-depth understanding of how these systems arrange in the solid state.

Data collection and refinement details for $[Pd_2\mathbf{D2}_4]^{4+}$

Yellow rod-shaped single crystals of $[Pd_2\mathbf{D2}_4]^{4+}$ were grown by vapor diffusion of benzene into a solution of $[Pd_2\mathbf{D2}_4]^{4+}$ in DMSO. A suitable crystal was selected and

mounted on a loop. Subsequently, the crystals were frozen in liquid N_2, placed in UNI Pucks and stored at cryogenic temperature in a dry shipper. UNI Pucks were transferred to the sample Dewar container, and all samples were mounted using a StäubliTX60L robotic arm. A wavelength of 1.0332 Å was chosen using a liquid N_2 cooled double crystal monochromator. Single crystal X-ray diffraction data was collected at 103(2) K on a single axis goniometer, equipped with an Oxford Cryostream 800 and Pilatus 6M detector. 1800 diffraction images were collected in a 360° φ sweep at a detector distance of 200 mm, 50% filter transmission, 0.1° step width and 100 milliseconds exposure time per image. Data integration and reduction were undertaken using XDS.[65] The resolution was cut off at 1.3083 Å.

Stereochemical restraints for organic building blocks (residue DOR) were generated by the GRADE program using the GRADE Web Server (http://grade.globalphasing.org) and applied in the refinement. The GRADE dictionary contains target values and standard deviations for 1,2-distances (DFIX) and 1,3-distances (DANG), as well as restraints for planar groups (FLAT). The anisotropic refinement for C, N, O atoms was enabled by a combination of similarity restraints (SIMU) and rigid bond restraints (RIGU).[71] The contribution of the electron density from solvent molecules, which could not be modelled with discrete atomic positions were handled using the SQUEEZE routine in PLATON. The program calculated a total solvent accessible volume/cell of 10130 Å^3 (47.4%), and a total electron-count/cell of 2540 electrons, likely due to the presence of sixteen of DMSO, twenty of benzene, and four of tetrafluoroborate. The solvent mask file (.fab) computed by PLATON was included in the SHELXL refinement via the ABIN instruction leaving the measured intensities untouched.

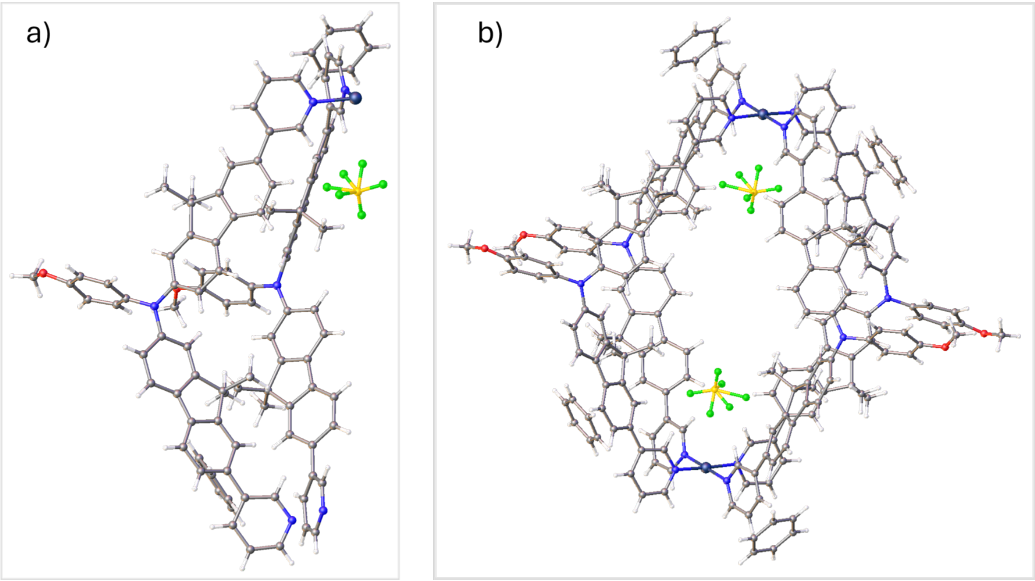

Fig. S37 a) Asymmetric unit and b) molecular structure of $[Pd_2\mathbf{D2}_4]^{4+}$. Color code: Pd dark blue, N blue, O red, C grey, B light yellow, F green, H white.

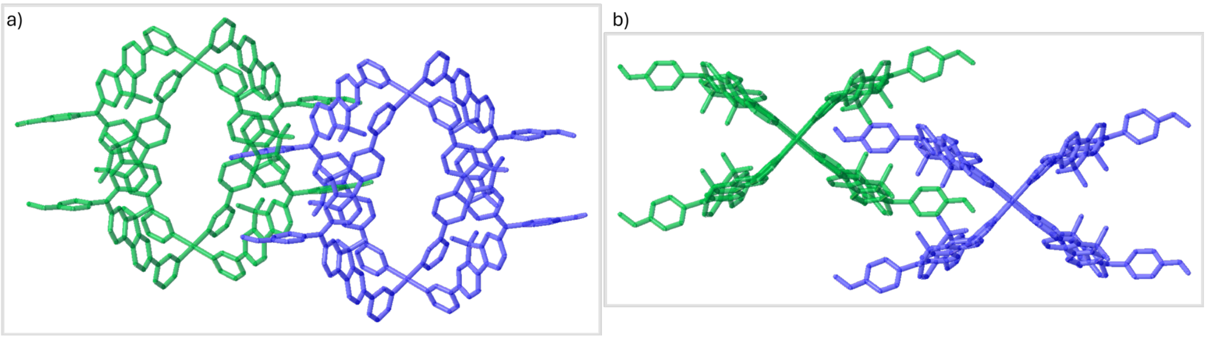

Fig. S38 a) Side view and b) top view of the packing between two $[Pd_2\mathbf{D2}_4]^{4+}$ with the ligand of one structure (green) stacked between two ligands of a second structure (purple).

Data collection and refinement details for $[Pd_2\mathbf{D2}_2\mathbf{A2}_2]^{4+}$

Yellow rod-shaped single crystals of $[Pd_2\mathbf{D2}_2\mathbf{A2}_2]^{4+}$ were grown by vapor diffusion of benzene into a solution of $[Pd_2\mathbf{D2}_2\mathbf{A2}_2]^{4+}$ in DMSO. A suitable crystal was selected and mounted on a loop. Subsequently, the crystals were frozen in liquid N_2, placed in UNI Pucks and stored at cryogenic temperature in a dry shipper. UNI Pucks were transferred to the sample Dewar container, and all samples were mounted using a StäubliTX60L robotic arm. A wavelength of 1.0332 Å was chosen using a liquid N_2 cooled double crystal monochromator. Single crystal X-ray diffraction data was collected at 103(2) K on a single axis goniometer, equipped with an Oxford Cryostream 800 and Pilatus 6M detector. 1800 diffraction images were collected in a 360° φ sweep at a detector distance of 200 mm, 0.1° step width and 100 milliseconds exposure time per image. Data integration and reduction were undertaken using XDS.[65] The resolution was cut off at 1.3001 Å.

Stereochemical restraints for organic building blocks (residue DOR, LA0) were generated by the GRADE program using the GRADE Web Server (http://grade.globalphasing.org) and applied in the refinement. The GRADE dictionary contains target values and standard deviations for 1,2-distances (DFIX) and 1,3-distances (DANG), as well as restraints for planar groups (FLAT). The anisotropic refinement for C, N, O atoms was enabled by a combination of similarity restraints (SIMU) and rigid bond restraints (RIGU).[71] The contribution of the electron density from solvent molecules, which could not be modelled with discrete atomic positions were handled using the SQUEEZE routine in PLATON. The program calculated a total solvent accessible volume/cell of 1214 Å^3 (16.9%), and a total electron-count/cell of 317 electrons, likely due to the presence of two of DMSO, four of benzene, and six of water. The solvent mask file (.fab) computed by PLATON was included in the SHELXL refinement via the ABIN instruction leaving the measured intensities untouched.

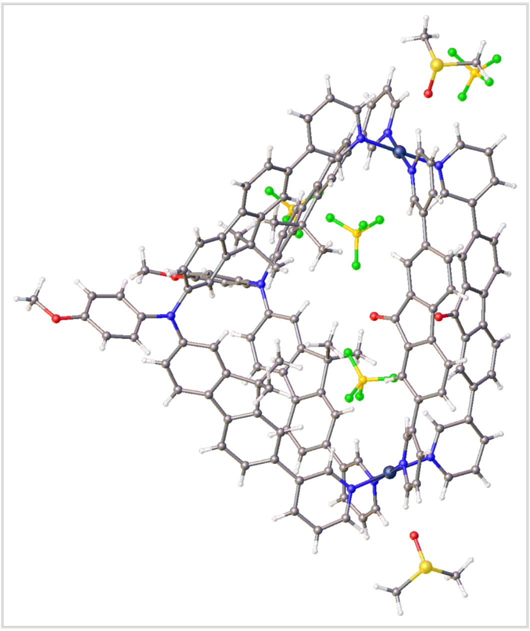

Fig. S39 Asymmetric unit of [Pd_2**D2**$_2$**A2**$_2$]$^{4+}$. Color code: Pd dark blue, N blue, O red, C grey, B light yellow, F green, S dark yellow, H white.

Tab. S1 Crystallographic data of [Pd_2**D2**$_4$]$^{4+}$ and [Pd_2**D2**$_2$**A2**$_2$]$^{4+}$.

	[Pd_2**D2**$_4$]$^{4+}$	[Pd_2**D2**$_2$**A2**$_2$]$^{4+}$
CCDC	2496389	2496390
Empirical formula	C212 H180 B2 F8 N12 O4 Pd2 + [C76 H108 B2 F8 O8 S8]	C144 H118 B4 F16 N10 O6 Pd2 S2 + [C14 H24 O4 S1]
Formula weight	3346.09	2708.64
Temperature	103(2)	103(2)
Wavelength	1.0332	1.0332
Crystal system	Trigonal	Triclinic
Space group	P-3	P-1
a (Å)	37.913(5)	17.676(4)
b (Å)	37.913(5)	21.489(4)
c (Å)	17.180(3)	22.471(5)
α (°)	-	118.27(3)
β (°)	-	91.17(3)
γ (°)	-	104.07(3)
Volume (Å^3)	21386(7)	7200(3)
Z	3	2
Density (calculated) (Mg/m^3)	0.779	1.249
Absorption coefficient (mm^{-1})	0.447	0.987
F(000)	5226	2776
Crystal size (mm^3)	0.120 x 0.010 x 0.010	0.020 x 0.010 x 0.003
Theta range for data collection (°)	0.902 to 23.258	1.515 to 23.412
Index ranges	-28<=h<=28, -28<=k<=28, -13<=l<=13	-13<=h<=12, -16<=k<=16, -17<=l<=17
Reflections collected	64642	21241
Independent reflections	6629 [R(int) = 0.0990]	6412 [R(int) = 0.0889]
Completeness to theta	99.60% (theta = 23.412°)	93.30% (theta = 23.258°)

Max. and min. transmission	1.000 and 0.987	0.987 and 0.789
Refinement method	Full-matrix least-squares on F^2	Full-matrix least-squares on F^2
Data / restraints / parameters	6629 / 1234 / 1076	6412 / 1822 / 1657
Goodness-of-fit on F^2	1.094	1.902
Final R indices [I>2sigma(I)]	R1 = 0.0704, wR2 = 0.2245	R1 = 0.1561, wR2 = 0.4107
R indices (all data)	R1 = 0.0944, wR2 = 0.2523	R1 = 0.1921, wR2 = 0.4381
Extinction coefficient	n/a	n/a
Largest diff. peak and hole (e·Å^{-3})	0.319 and -0.293	2.639 and -0.592

4. Photophysical characterization

4.1. Steady state UV-Vis absorption spectroscopy

UV-Vis spectra were recorded on a DAD HP-8453 UV-Vis spectrometer. The step size was 1 nm and the cuvette pathlength was 10 mm.

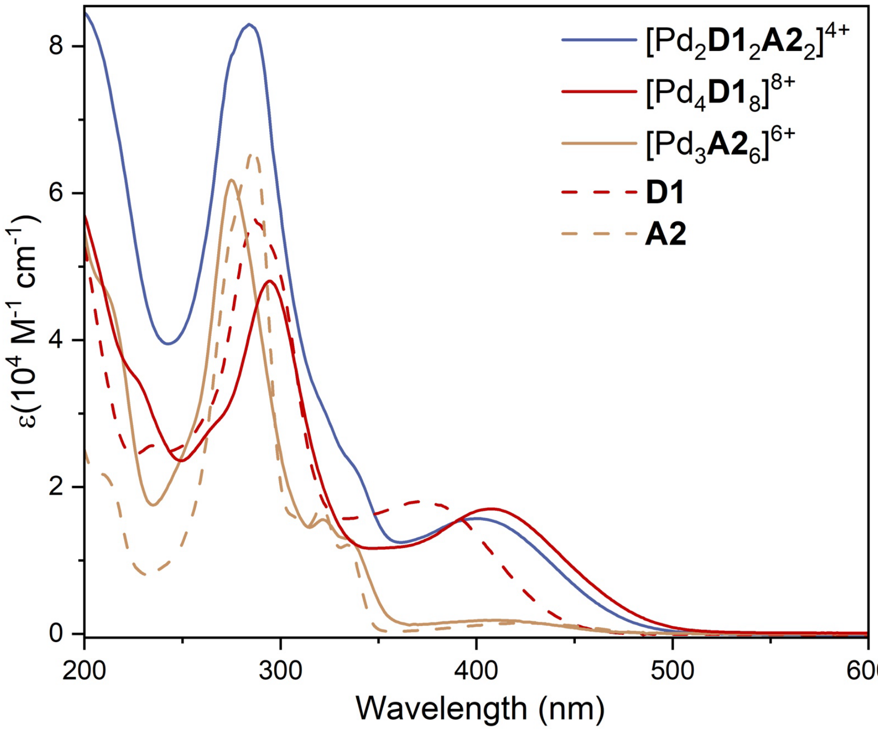

Fig. S40 UV-Vis absorption spectra of $[Pd_2\mathbf{D1}_2\mathbf{A2}_2]^{4+}$ and the respective homoleptic assemblies and ligands in CH_3CN.

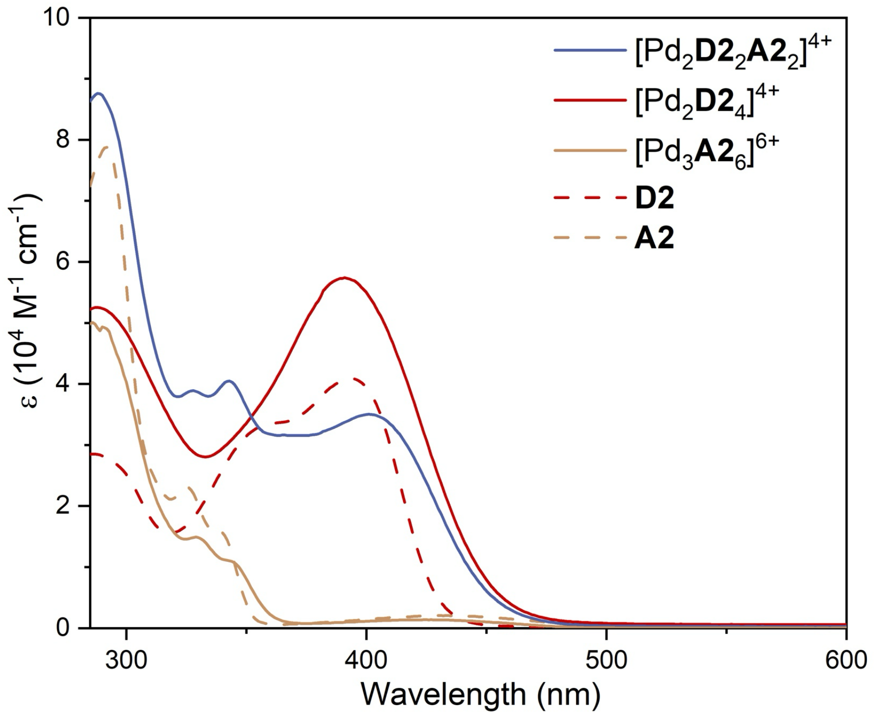

Fig. S41 UV-Vis absorption spectra of $[Pd_2\mathbf{D2}_2\mathbf{A2}_2]^{4+}$ and the respective homoleptic assemblies and ligands in DMSO.

4.2. Steady state emission spectroscopy

Fluorescence spectra were recorded on a Jasco FP-8300 fluorimeter. The cuvette pathlength was 10 mm and the step size 0.5 nm. The fluorescence spectra of the ligands and the cages were compared in order to investigate emission quenching via the population of dark states such as charge transfer states. The cage self-assembly is an equilibrium reaction meaning that "free" (solvent-coordinated) Pd(II) ions and ligands can in principle always be present in solution. Dilution of the sample shifts the equilibrium to the free ligands and Pd(II). This leads to two main problems when measuring the photophysical properties of the system: *i*) the formation of the cage is confirmed by NMR at a rather high concentration, and where small amounts of free ligand might not be visible due to the low sensitivity of this technique; *ii*) diluting the compound results in a release of free ligand and its emission can cover possible emission bands of the cage. To prevent this problem, it is possible to add an excess of the Pd(II) salt for pushing the equilibrium to the formation of the cages, even at low

ligand concentrations. This effect was studied for the donor-acceptor cage $[Pd_2\mathbf{D2}_2\mathbf{A2}_2]^{4+}$. Comparison of the emission spectra at concentrations of 10^{-3}, 10^{-4}, and 10^{-5} M (ligand concentration) with a stoichiometric amount of Pd(II) shows that the emission for the compounds at higher concentration is essentially quenched. On the other hand, the diluted sample (10^{-5} M) shows a strong emission with the same maximum and shape as free ligand **D2** (Fig. S42 and S43). Addition of an excess of the Pd(II) salt leads to quenching which further supports that the observed emission stems from traces of free ligand (Fig. S44). Interestingly, the residual emission of the 10^{-4} and 10^{-5} M samples resemble the shape of the emission of free ligand **D2**, while the emission of the 10^{-3} M sample has the same shape as compared to the homoleptic assembly of ligand **A2** which keeps its emission upon coordination to Pd(II).[49] Presumably, this is then due to small amounts of free ligand **D2** or ring $[Pd_3\mathbf{A2}_6]^{6+}$ in the solution, respectively. We conclude from these experiments that the emission of the donor-acceptor cage $[Pd_2\mathbf{D2}_2\mathbf{A2}_2]^{4+}$ is quenched. A similar conclusion was drawn for the emission of the homoleptic cage $[Pd_2\mathbf{D2}_4]^{4+}$ (Fig. S42).

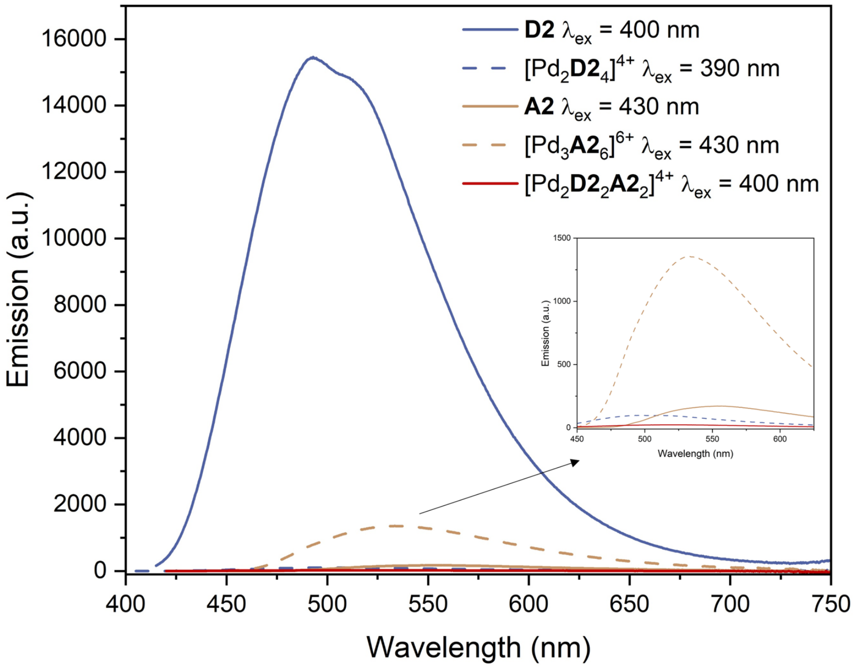

Fig. S42 Emission spectra of $[Pd_2\mathbf{D2}_2\mathbf{A2}_2]^{4+}$ and the respective homoleptic assemblies and ligands (DMSO, $c = 1{\cdot}10^{-5}$ M, the here seen smaller emission of free **A2** as compared to blue-shifted $[Pd_3\mathbf{A2}_6]^{6+}$ [compare ref. 49] may have resulted from aggregation in the sample of free ligand).

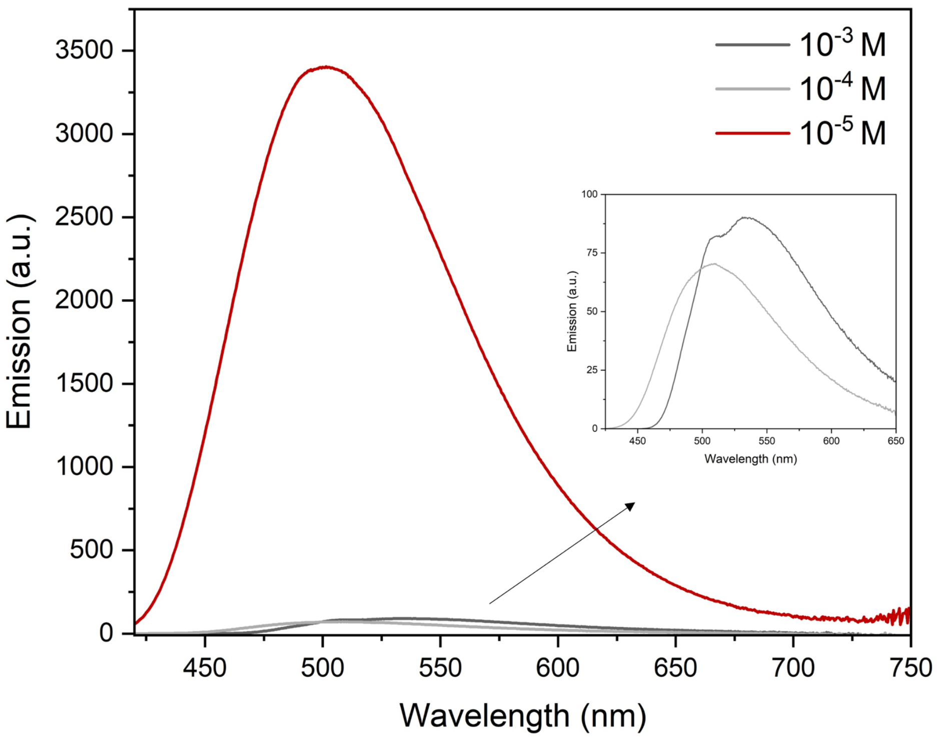

Fig. S43 Emission spectra of the cage $[Pd_2\mathbf{D2}_2\mathbf{A2}_2]^{4+}$ at different concentrations (DMSO, concentration given as ligand concentration, λ_{ex} = 400 nm). No excess of $[Pd(CH_3CN)_4](BF_4)_2$ added.

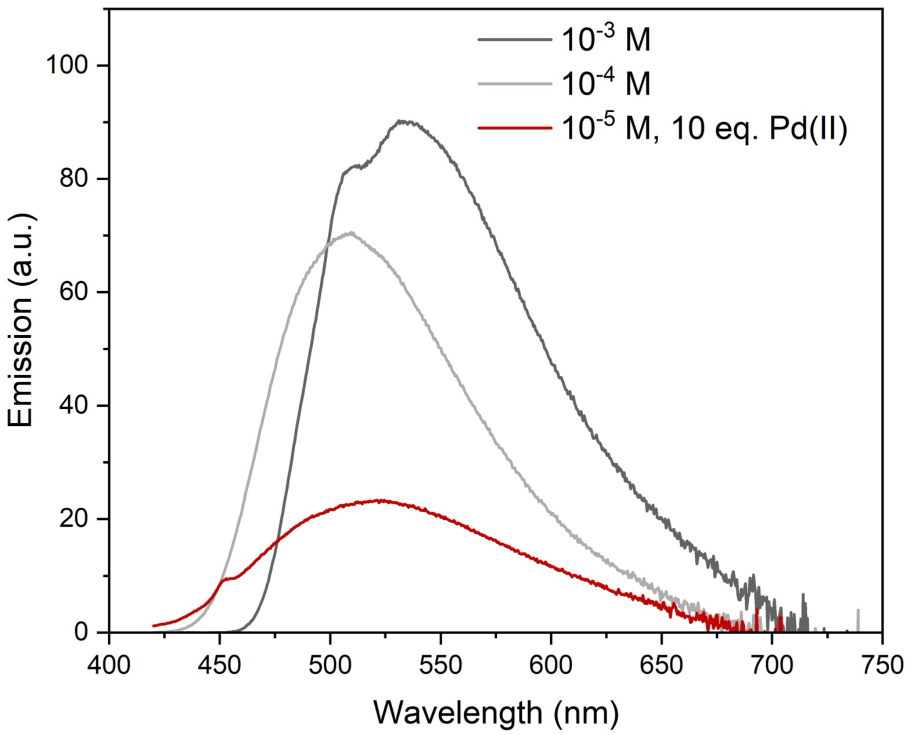

Fig. S44 Emission spectra of the cage $[Pd_2\mathbf{D2}_2\mathbf{A2}_2]^{4+}$ at different concentrations (DMSO, concentration given as ligand concentration, λ_{ex} = 400 nm). 10 equivalents of $[Pd(CH_3CN)_4](BF_4)_2$ are used for the sample at $1.4{\cdot}10^{-5}$ M to ensure the almost quantitative formation of the heteroleptic cage.

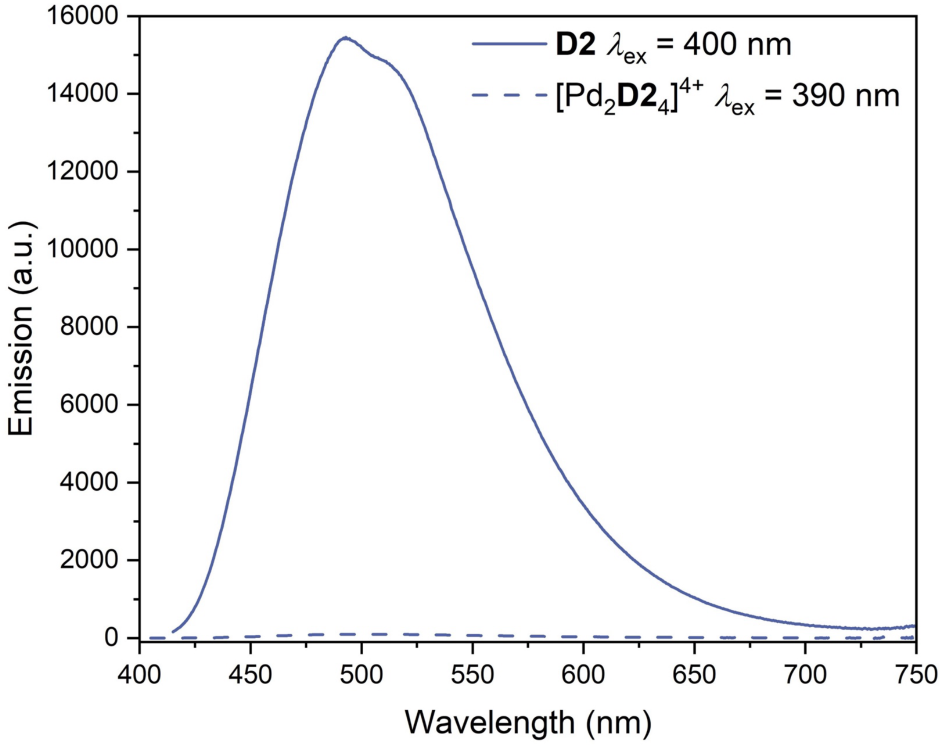

Fig. S45 Emission spectra of ligand **D2** and homoleptic cage $[Pd_2\textbf{D2}_4]^{4+}$ (DMSO, *c* = $1{\cdot}10^{-5}$ M)

4.3. Steady state IR absorption spectroscopy

For acquisition of the Fourier-transform infrared (FTIR) spectra, a Perkin Elmer SpectrumTwo FTIR spectrometer was used. The solid samples (ligands) were measured in the reflection mode using a diamond attenuated total reflection (ATR) unit. The liquid samples were measured in transmittance mode using a liquid holder cell mount, with a spacer of 0.1 mm in between two KCl glasses.

For details on the IR spectra calculations see section 6.3.

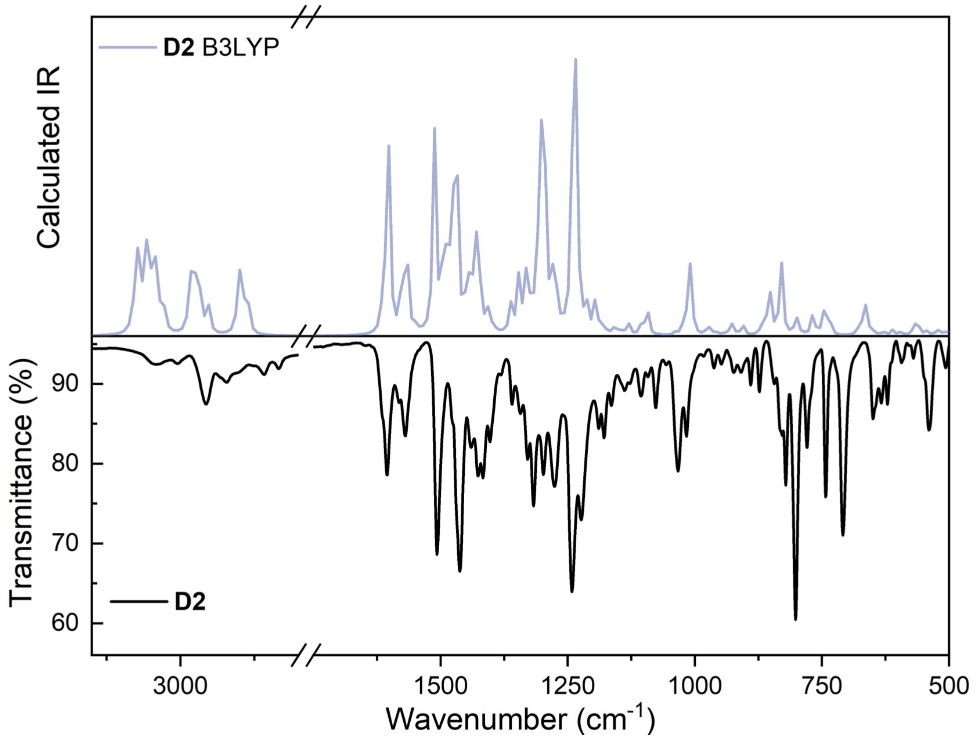

Fig. S46 IR spectra of the free donor ligand **D2**; top: calculated spectrum (method: B3LYP/6-31g++(d,p) with implicit solvent);[61] bottom: experimental ATR spectrum in CH_3CN.

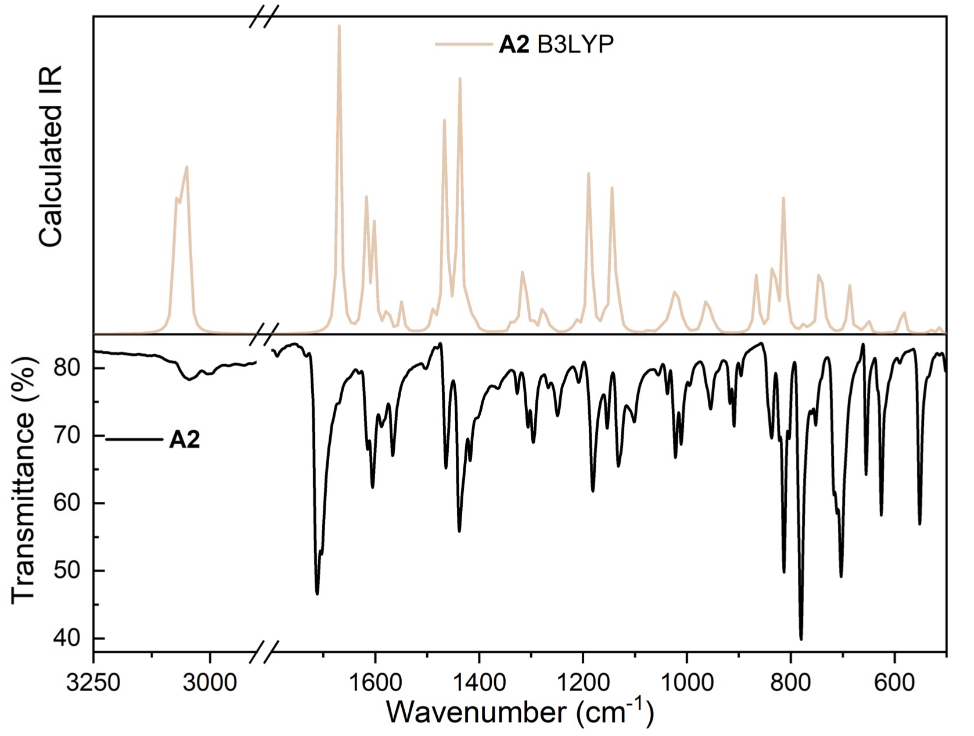

Fig. S47 IR spectra of the free acceptor ligand **A2**; top: calculated spectrum (method: B3LYP/6-31g++(d,p) with implicit solvent);[61] bottom: experimental ATR spectrum in CH_3CN.

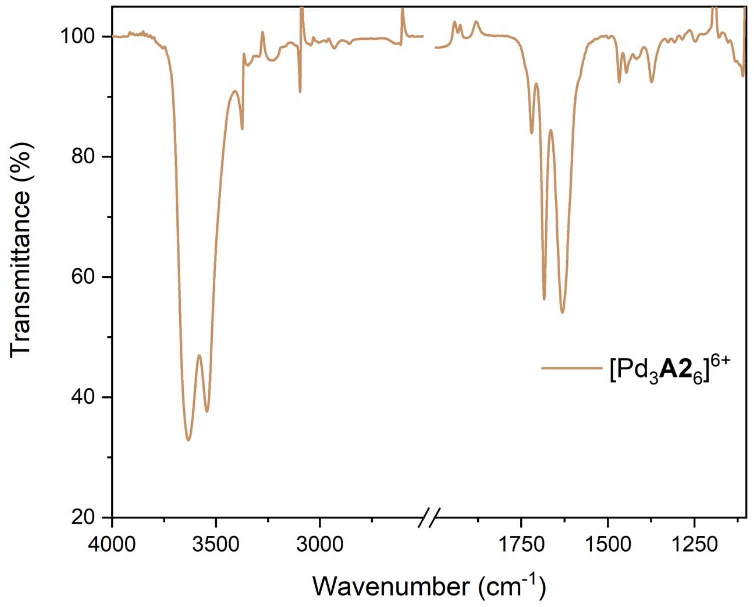

Fig. S48 IR spectrum of the homoleptic assemblies of ligand **A2** ($[Pd_3\mathbf{A2}_6]^{6+}$ + $[Pd_4\mathbf{A2}_8]^{8+}$, simplified as $[Pd_3\mathbf{A2}_6]^{6+}$ in the figure).

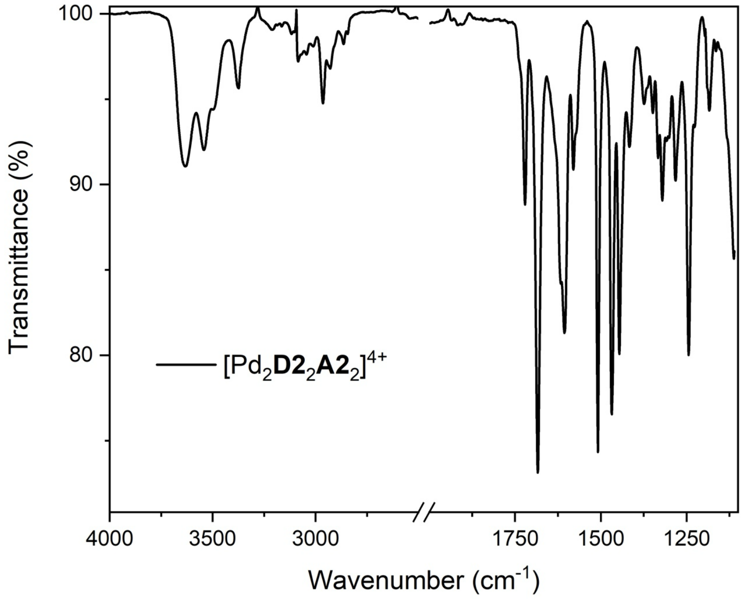

Fig. S49 IR spectrum of the donor-acceptor cage $[Pd_2\mathbf{D2}_2\mathbf{A2}_2]^{4+}$.

5. Electrochemistry

5.1. Cyclic voltammetry

Cyclic voltammograms were recorded using a Metrohm potentiostat PGSTAT101 and the NOVA electrochemistry software (Version 1.9) included in Metrohm Autolab. If not mentioned otherwise, a glassy carbon working electrode and a platinum wire counter electrode were used. The working electrode was polished regularly between the

measurements using a polishing powder. As reference electrode Ag/AgNO3 (in CH_3CN or DMSO with 0.1 M $TBAPF_6$) was used. All measurements were performed at room temperature. The samples were degassed via purging with inert gas before scanning at negative potentials. The samples were dissolved in the non-aqueous electrolyte solution (CH_3CN or DMSO with 0.1 M $TBAPF_6$) prior to the measurement. The cyclic voltammograms were smoothed using Origin 2023 software.

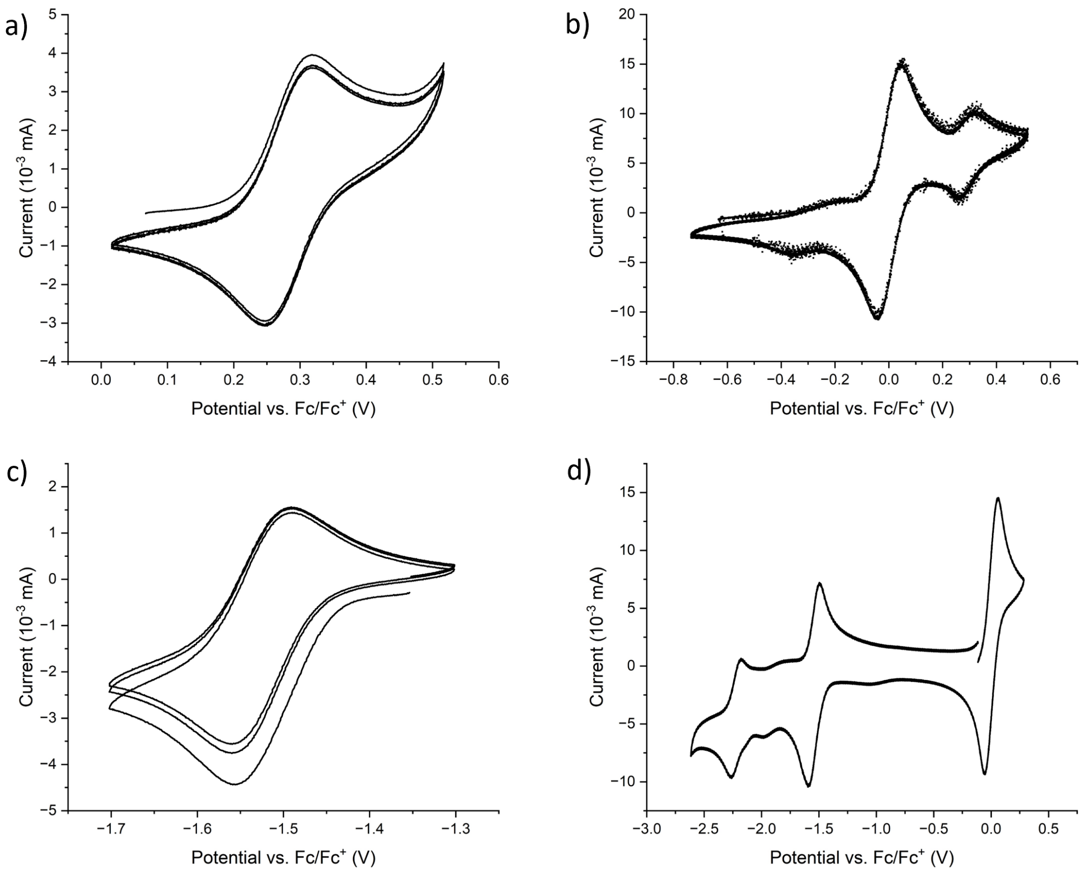

Fig. S50 Cyclic voltammograms of ligands: a), b) ligand **D2**, c), d) ligand **A2** (1.4 mM, DMSO, 0.1 M $TBAPF_6$, WE: glassy carbon, CE: platinum wire, RE: Ag/$AgNO_3$, scan rate: 0.1 $V{\cdot}s^{-1}$). The cyclic voltammograms are referenced to the ferrocene-ferrocenium couple (event at 0 V).

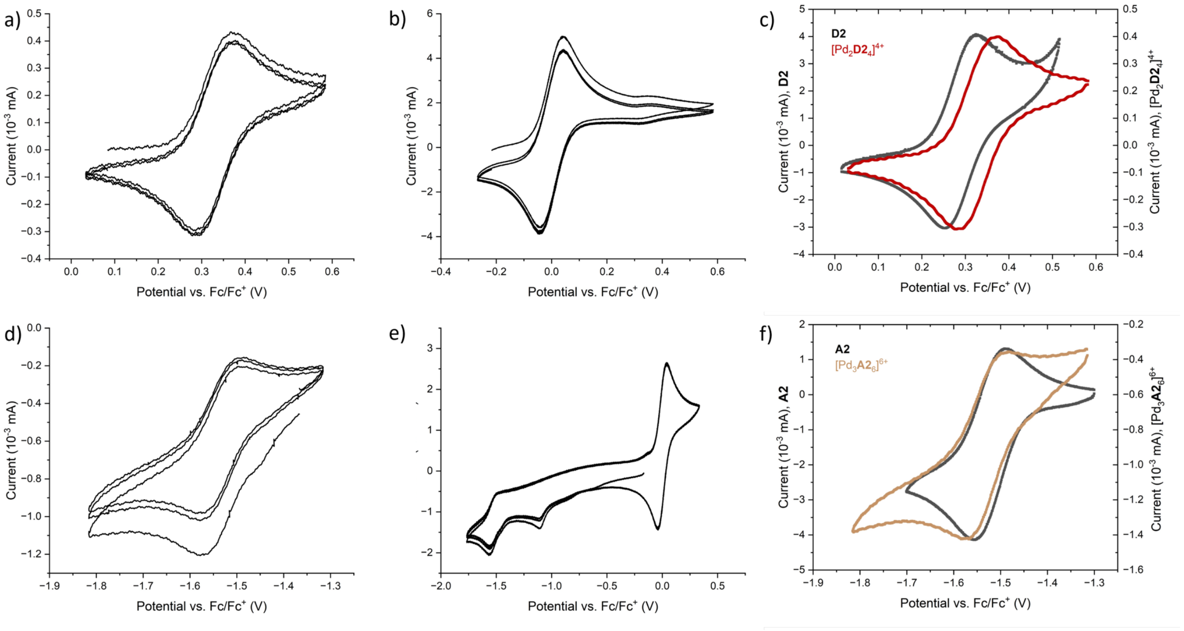

Fig. S51 Cyclic voltammograms of homoleptic assemblies: a), b) homoleptic cage [Pd_2**D2**$_4$]$^{4+}$, c) ligand **D2** and cage [Pd_2**D2**$_4$]$^{4+}$ in comparison, d), e) homoleptic assembly [Pd_3**A2**$_6$]$^{6+}$, f) ligand **A2** and homoleptic assembly [Pd_3**A2**$_6$]$^{6+}$ in comparison (1.4 mM, DMSO, 0.1 M $TBAPF_6$, WE: glassy carbon, CE: platinum wire, RE: Ag/$AgNO_3$, scan rate: 0.1 V·s^{-1}). The cyclic voltammograms are referenced to the ferrocene-ferrocenium couple (event at 0 V).

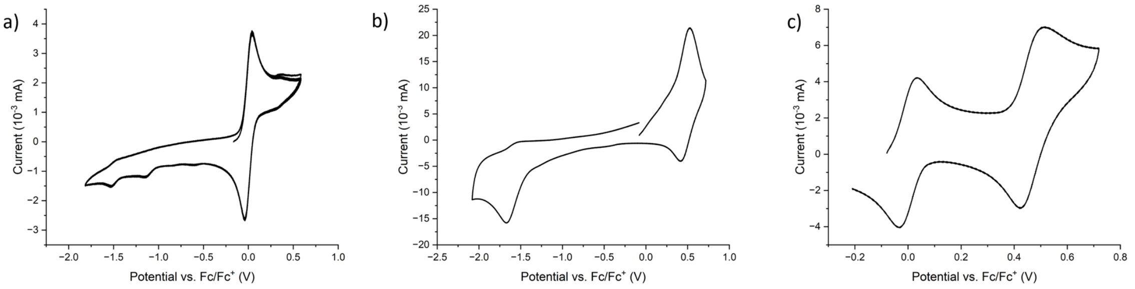

Fig. S52 Cyclic voltammograms of donor-acceptor cages: a) [Pd_2**D2**$_2$**A2**$_2$]$^{4+}$ (1.4 mM, DMSO), b) and c) [Pd_2**D2**$_2$**A2**$_2$]$^{4+}$ (c = 160 µM, CH_3CN); (0.1 M $TBAPF_6$, WE: glassy carbon, CE: platinum wire, RE: Ag/$AgNO_3$, scan rate: 0.1 V·s^{-1}). The cyclic voltammograms are referenced to the ferrocene-ferrocenium couple (event at 0 V).

Tab. S2 Half-wave potentials $E_{1/2}$ of the ligands, their homoleptic assemblies, and the donor-acceptor cages. The potentials are given in V and are referenced to Fc/Fc$^+$.[1] in CH_3CN, [2] in DMSO.

	**D1**	**D2**	**A2**
Free ligand	0.39[33],1	0.29[2]	−1.53[2]
Homoleptic assembly $[Pd_nD_{2n}]^{2n+}$	0.52 (n = 4)[33],1	0.33 (n = 2)[2]	−1.52[2]
$[Pd_2$**D1**$_2$**A2**$_2]^{4+}$	0.47[1]	-	−1.60[1]
$[Pd_2$**D2**$_2$**A2**$_2]^{4+}$	-	0.34[2]	−1.49[2]

Based on the spectroscopic, electrochemical, and computational data, the thermodynamic driving force for photoinduced electron transfer can be estimated by applying equation 1:[72]

$$\Delta G_{PET} = -e \cdot (E_{red} - E_{ox}) - E_{00} - E_C \quad (1)$$

E_{00} corresponds to the energy of the donor's excited singlet state and is obtained from the intersection of the donor's absorption and emission spectrum. E_{red} and E_{ox} are the half-wave potentials and are obtained from the cyclic voltammogram (Tab. S2) or from the DFT computations (only for the LUMO located on the Pd(II) node, Tab. S4). E_C is the Coulomb-term ($\frac{e^2}{4\pi\varepsilon_0\varepsilon_r d_{DA}}$), accounting for the electrostatic attraction of the formed radical ions; for an estimated distance of 10 Å, the value amounts to 0.04 eV in CH_3CN.

Tab. S3 Free energy of photoinduced electron transfer from the photoexcited donor ligand D* to the Pd(II) node or the acceptor ligand A.

	ΔG_{PET}(D*→Pd(II))	ΔG_{PET}(D*→A)
$[Pd_2$**D1**$_2$**A2**$_2]^{4+}$	−0.18	−0.67
$[Pd_2$**D2**$_2$**A2**$_2]^{4+}$	−0.54	−1.13

5.2. Spectroelectrochemistry

For the spectroelectrochemical studies, a thin layer quartz glass spectroelectrochemical cell kit from ALS Japan was used. The optical path length was either 0.5 mm or 1 mm.

A gold (for d = 0.5 mm) or a platinum mesh electrode (for d = 1 mm) was used as working electrode. A platinum wire was used as counter electrode and a non-aqueous Ag/$AgNO_3$ (in CH_3CN or DMSO with 0.1 M $TBAPF_6$) as reference electrode. The compounds were oxidized or reduced by bulk electrolysis using the potentiostat PGSTAT101 and the NOVA electrochemistry software (Version 1.9) included in Metrohm Autolab. For the UV-Vis absorption measurements, an AvaLight Deuterium-Halogen light source (200 nm - 1000 nm) was used. For the conduction of the light to the spectroelectrochemical cell and to the DAD AVA-SPEC 2048 spectrometer, an optical fiber with a diameter of 200 µm was used. The data were recorded using AVASOFT 7.5 software. The spectra were smoothed using Origin 2023 software. Before the application of negative potentials, the sample was degassed via purging with inert gas. The samples were dissolved in the non-aqueous electrolyte solution (CH_3CN or DMSO with 0.1 M $TBAPF_6$) prior to the measurement.

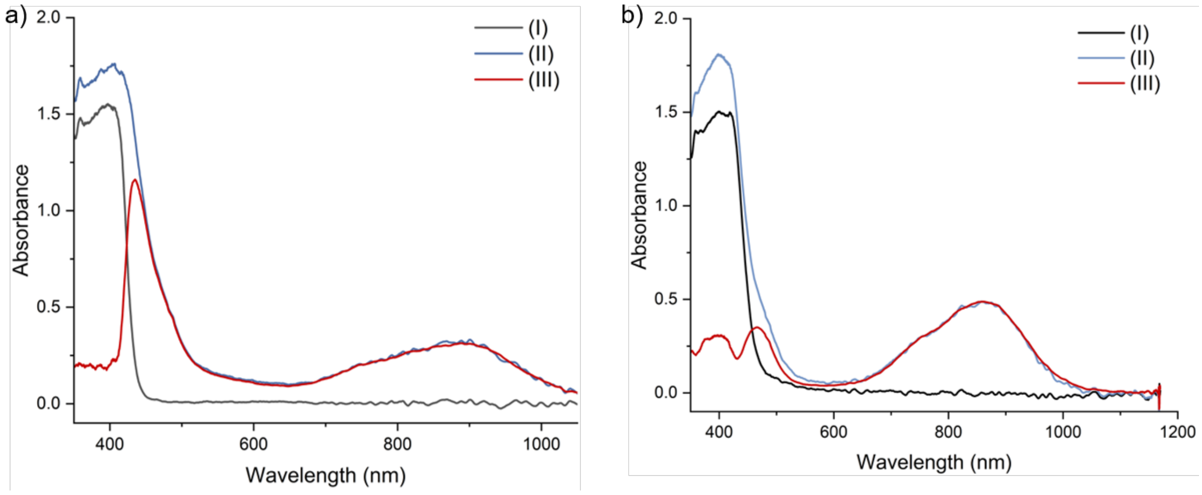

Fig. S53 UV-Vis absorption spectra recorded upon electrochemical oxidation of ligand **D2** (a) and homoleptic $[Pd_2\mathbf{D2}_4]^{4+}$ (b). (I) ground state, (II) upon application of a potential of +0.4 V, (III) difference spectrum (c = 1.4 mM, DMSO, 0.1 M $TBAPF_6$, WE: platinum gauze, CE: platinum wire, RE: Ag/$AgNO_3$, cuvette pathlength: 1.0 mm).

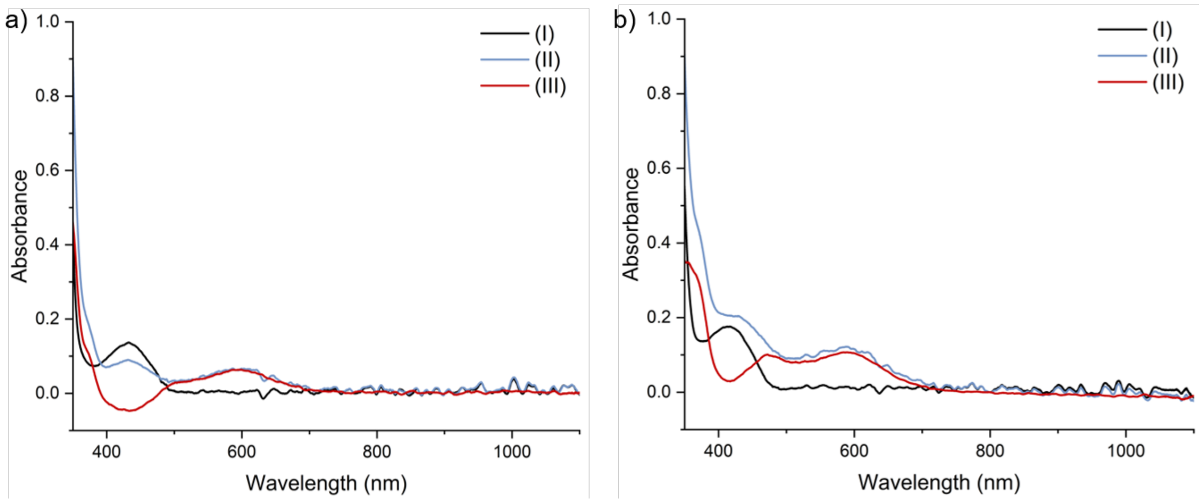

Fig. S54 UV-Vis absorption spectra recorded upon electrochemical oxidation of ligand **A2** (a) and homoleptic $[Pd_3\mathbf{A2}_6]^{6+}$ (b). (I) ground state, (II) upon application of a potential of −1.6 V, (III) difference spectrum (c = 1.4 mM, DMSO, 0.1 M $TBAPF_6$, WE: platinum gauze, CE: platinum wire, RE: $Ag/AgNO_3$, cuvette pathlength: 1.0 mm).

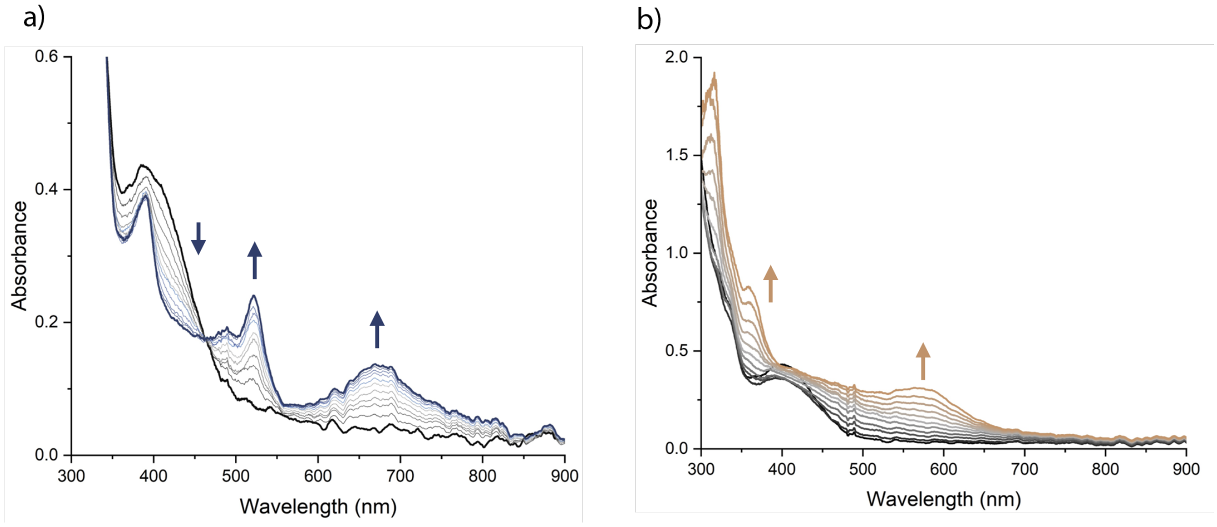

Fig. S55 UV-Vis absorption spectra of $[Pd_2\mathbf{D1}_2\mathbf{A2}_2]^{4+}$ recorded upon oxidation (a), *E* = +0.60 V) and reduction (b), *E* = −1.80 V); steady state spectra are shown in black, the spectra of oxidized and reduced cage in blue and beige, respectively (*c*(cage) = 160 μM, CH_3CN, 0.1 M $TBAPF_6$, WE: gold gauze, CE: platinum wire, RE: $Ag/AgNO_3$, cuvette pathlength: 0.5 mm).

6. Computational studies

6.1. Binding angles

The binding angle corresponds here to the angle between the two nitrogen donor vectors (vector from C4 to N1 in the pyridine) of the ligands. All herein reported ligands

are equipped with *meta*-pyridine donor sites. Those ligands can adopt a variety of different binding angles via rotation around the backbone-pyridine bond. The binding angle was derived based on a ligand conformation in which the two pyridines have a similar orientation relative to the backbone. For this, two boundary cases exist, the concave and the convex binding mode.[49] The binding angle of the concave mode is smaller, hence leading to entropically favored smaller assemblies and this binding mode was considered here (Fig. S56). For the cages, the binding angles were calculated based on the geometry-optimized cages (section 6.2.) or based on the X-ray crystal structures, when available.

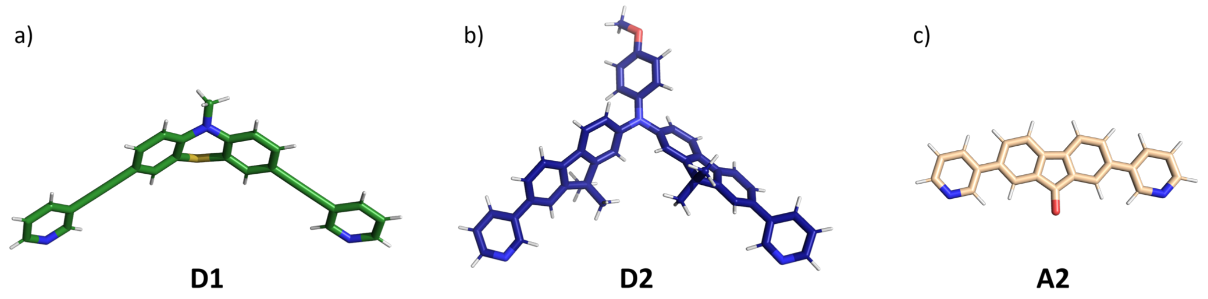

Fig. S56 Ligand conformations used for the determination of the binding angle.

6.2. Geometry optimizations and energy calculations

Models of the donor-acceptor cages $[Pd_2\mathbf{D1}_2\mathbf{A2}_2]^{4+}$ and $[Pd_2\mathbf{D2}_2\mathbf{A2}_2]^{4+}$ were built in the software Spartan[73] and pre-geometry optimized using the semiempirical PM6 method. This was followed by geometry optimization via DFT-computations (B3LYP/Def2-SVP) and single point energy calculations (B3LYP/Def2-TZVP) using Gaussian 16 software.[74] The energy calculations were carried out using CPCM implicit solvent model (acetonitrile). The orbitals were visualized with ChemCraft 1.8.[75]

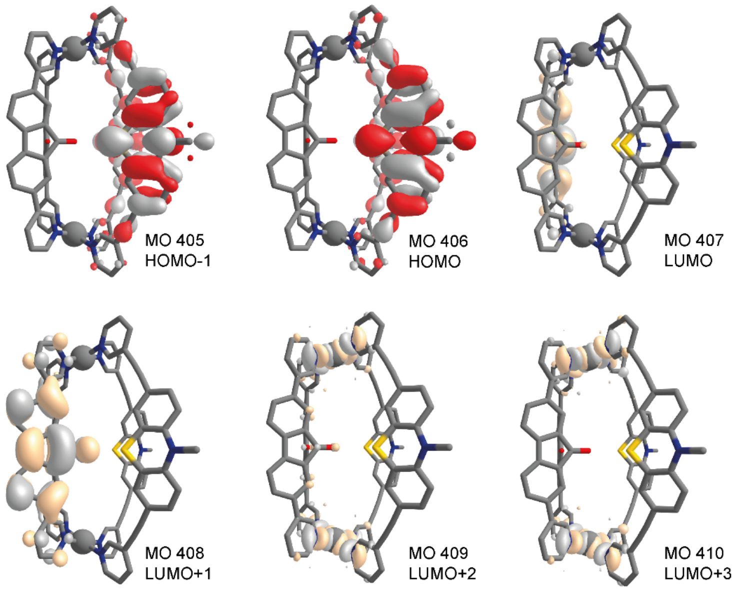

Fig. S57 Selected molecular orbitals of $[Pd_2\mathbf{D1}_2\mathbf{A2}_2]^{4+}$.

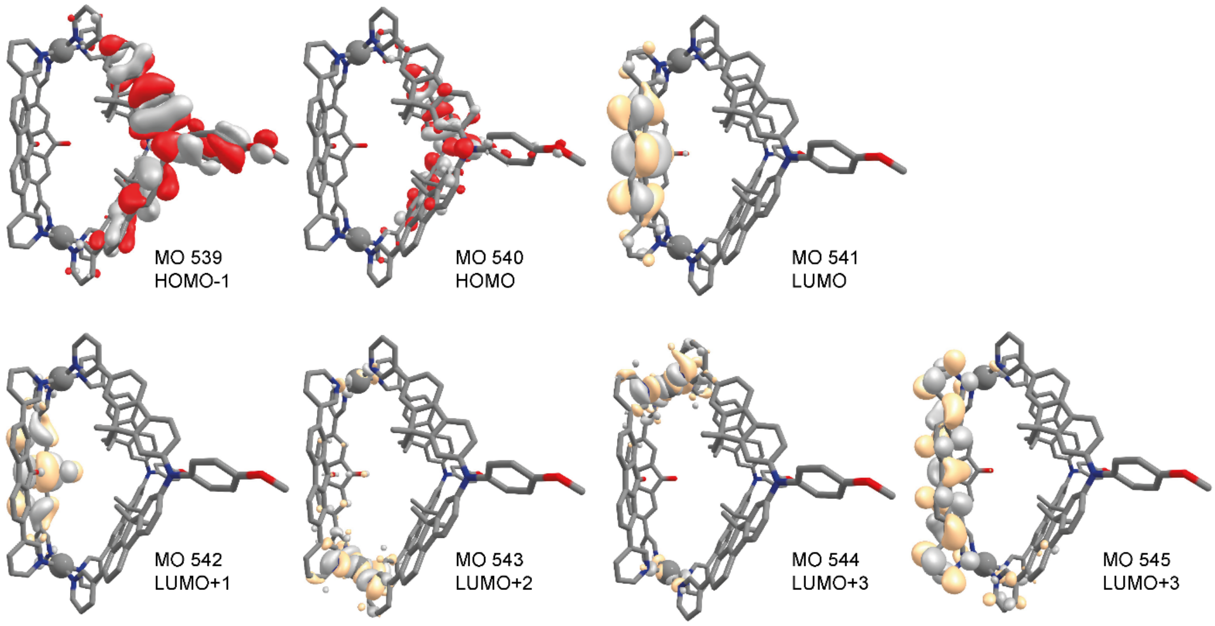

Fig. S58 Selected molecular orbitals of $[Pd_2\mathbf{D2}_2\mathbf{A2}_2]^{4+}$.

Tab. S4 Overview of experimentally and computationally determined frontier orbital energy levels of the donor-acceptor cages. $E_{HOMO/LUMO}$ = $-E_{1/2}-4.8$ eV, single point energy calculation: B3LYP/Def2-TZVP (CPCM solvent model, CH_3CN).

	$E_{HOMO,exp}$ (eV)	$E_{LUMO,exp}$ (eV)	$E_{HOMO,DFT}$ (eV)	$E_{LUMO,DFT}$ (eV)	$E_{LUMO(Pd),DFT}$ (eV)
$[Pd_2\mathbf{D1}_2\mathbf{A2}_2]^{4+}$	-5.27	-3.20	-5.66	-3.21	-2.71
$[Pd_2\mathbf{D2}_2\mathbf{A2}_2]^{4+}$	-5.14	-3.31	-5.24	-3.20	-2.72

6.3. Calculation of IR absorption spectra

The calculated IR spectra of the ligands and relative radical cation (for **D2**) and anion (for **A2**) was performed using Gaussian 16.[76] First a geometry optimization was obtained utilizing a B3LYP/6-31g++(d,p) functional and basis set, followed by a frequency calculation with the same basis set and implicit solvent. Next, a correction factor of 0.975 was applied to the calculated IR spectrum, to match the experimentally determined data (in the case of the ligands) following a literature reference.[61]

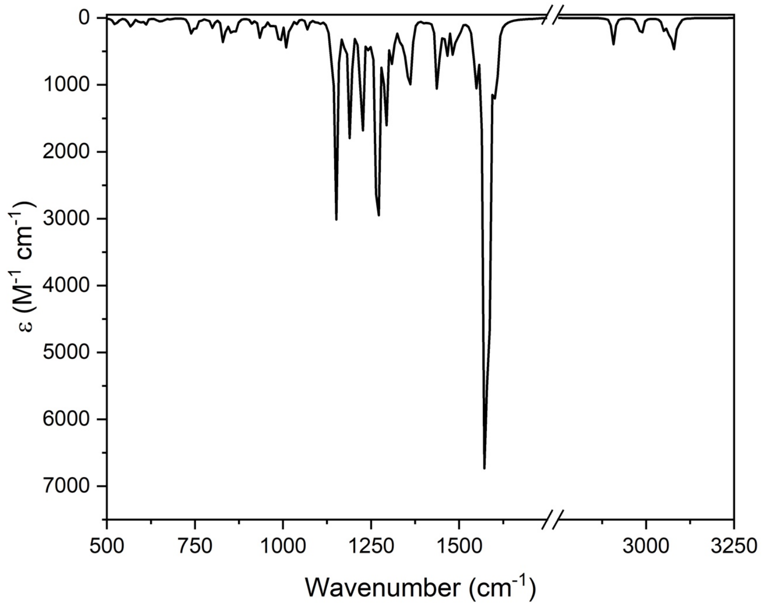

Fig. S59 Calculated IR absorption spectrum of the radical cation of donor ligand **D2**.

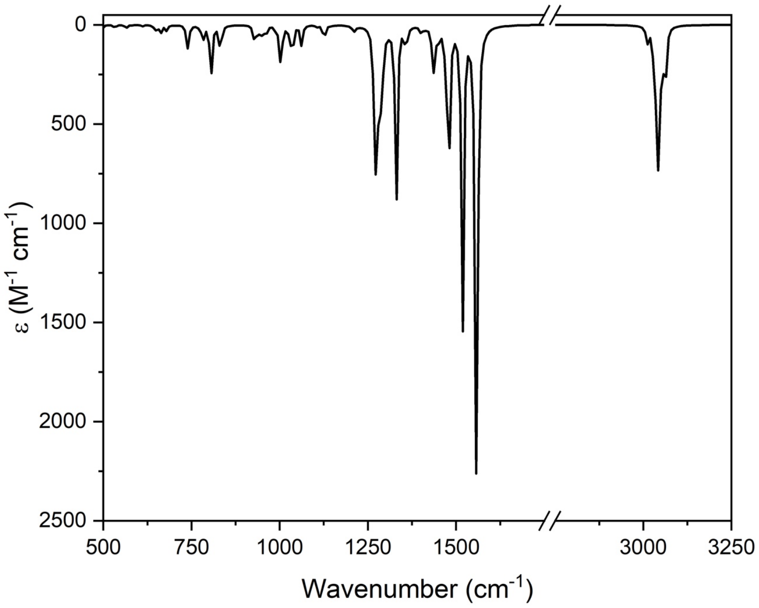

Fig. S60 Calculated IR absorption spectrum of the radical anion of acceptor ligand **A2**.

7. Transient absorption spectroscopy

A 1 kHz Ti:sapphire oscillator/regenerative amplifier (RGA) system (Solstice Ace, Spectra Physics) producing 4mJ/35 fs pulses at 800 nm was used to perform transient UV/Vis pump-probe experiments. Pump pulses at a wavelength of 375 nm were generated by using 3.5 mJ of the 800 nm energy to pump an optical parametric amplifier with subsequent fourth harmonic generation (TOPAS Prime+, Light Conversion). Pump pulses at 400 nm were produced by second harmonic generation of the RGA output. A fraction of ~2 µJ of the pump pulse energy focused to a diameter of about 200 μm was used to excite the sample. The probe pulse consisted of a white-light continuum generated by focusing ~1% of the 800 nm light into a 4 mm thick sapphire crystal. About 30% of white light served as a reference, the other 70% was overlapped with the pump pulse at the sample. Every 2nd pump pulse was blocked with a synchronized chopper to measure difference spectra induced by the pump pulse. The relative polarization angle between pump and probe pulse was adjusted to 54.7° to avoid signal contributions arising from rotational relaxation of the anisotropic distribution of excited molecules. Spectra were recorded in the wavelength range 350-

730 nm by means of two spectrographs (for reference and probe light) each equipped with a 256 element linear diode array. Time delays between pump and probe pulses were adjusted with a motorized translation stage (Newport Model DL325). The sample solutions were kept in a quartz glass cell that had an optical path length of 2 mm. Accumulation of photoproducts in the pump laser focus was avoided by use of a magnetic stirrer. Data acquisition was controlled with a LabVIEW program. The recorded spectra were corrected with respect to time shifts introduced by group delay dispersion within the white light probe continuum.

Transient IR absorption spectroscopy was performed with an analogous 1 kHz Ti:sapphire oscillator/RGA system (Coherent, Libra) producing 100 fs pulses at 800 nm. Part of the RGA output was used to generate pump pulses at 400 nm by second harmonic generation. Another portion (0.6 mJ) was utilized to pump a home-built two-stage optical parametric amplifier with subsequent difference frequency mixing to produce tunable IR probe pulses with a bandwidth of about 200 cm^{-1}. The IR light was split into a reference and a probe beam. The latter passed a motorized translation stage, which allowed adjusting the pump-probe time delay up to 1.8 ns. The pump energy was typically 2 μJ focused to a diameter of 200 μm at the sample. The sample cell consisted of stainless steel equipped with CaF_2 windows and a magnetic stirrer.[77] After passing the sample cell reference and probe IR pulses were dispersed in a spectrograph and recorded with a liquid nitrogen cooled HgCdTe-detector (Infrared Associates Inc.) consisting of 2 linear arrays of 32 pixel. The mid IR beam path was purged with dry nitrogen to minimize pulse distortions in air caused by CO_2 and water absorptions.

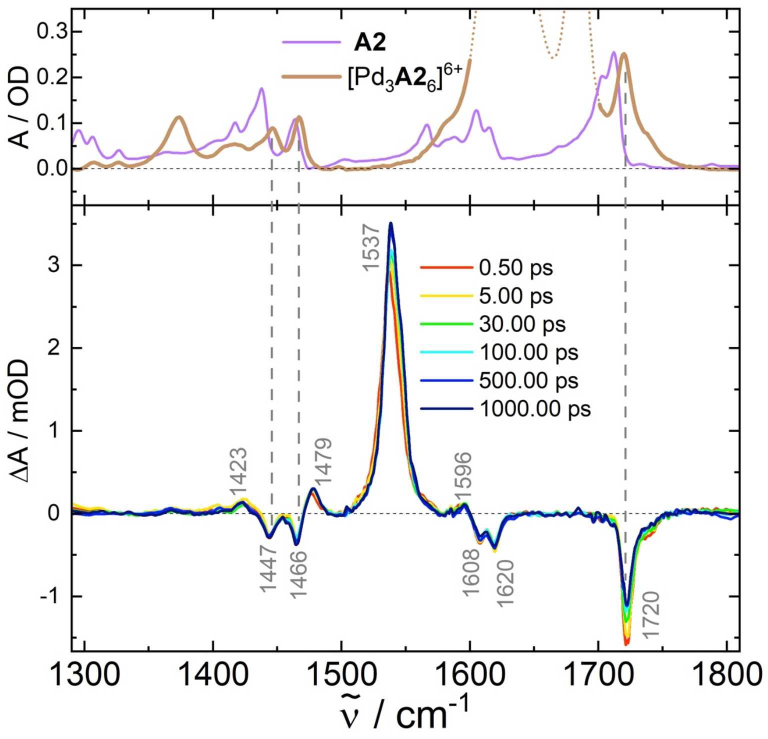

Fig. S61 Transient IR difference spectra of the homoleptic assemblies of ligand **A2** ($[Pd_3$**A2**$_6]^{6+}$ + $[Pd_4$**A2**$_8]^{8+}$) at various delays following 400 nm excitation (lower panel). The upper panel shows stationary FTIR spectra of $[Pd_3$**A2**$_6]^{6+}$ + $[Pd_4$**A2**$_8]^{8+}$ in CH_3CN (beige, simplified as $[Pd_3$**A2**$_6]^{6+}$ in the figure; the dotted region is distorted by an impurity) and ligand **A2** (violet, ATR).

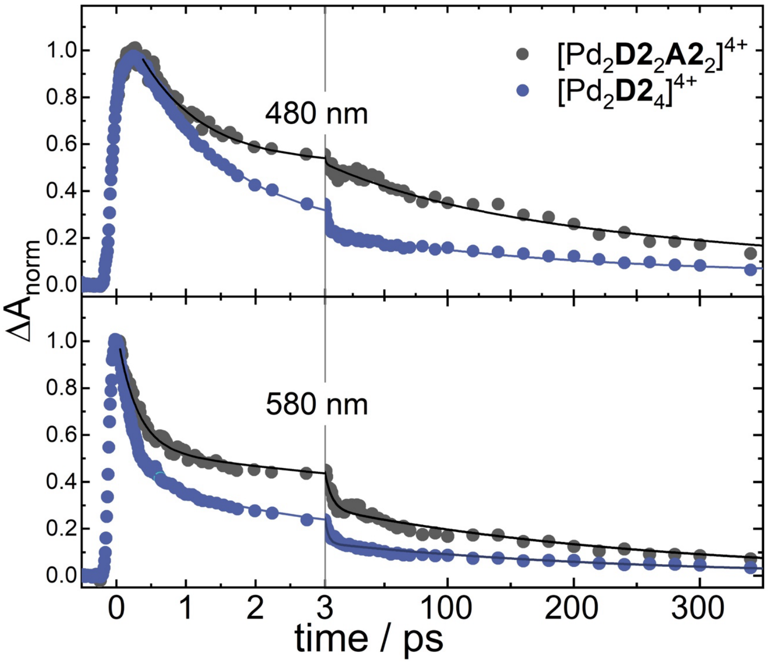

Fig. S62 Normalized time traces for the homoleptic cage $[Pd_2$**D2**$_4]^{4+}$ (blue) and the donor-acceptor cage $[Pd_2$**D**$_2$**A**$_2]^{4+}$ (black) at probe wavelength 480 (upper panel) and 580 nm (lower panel).

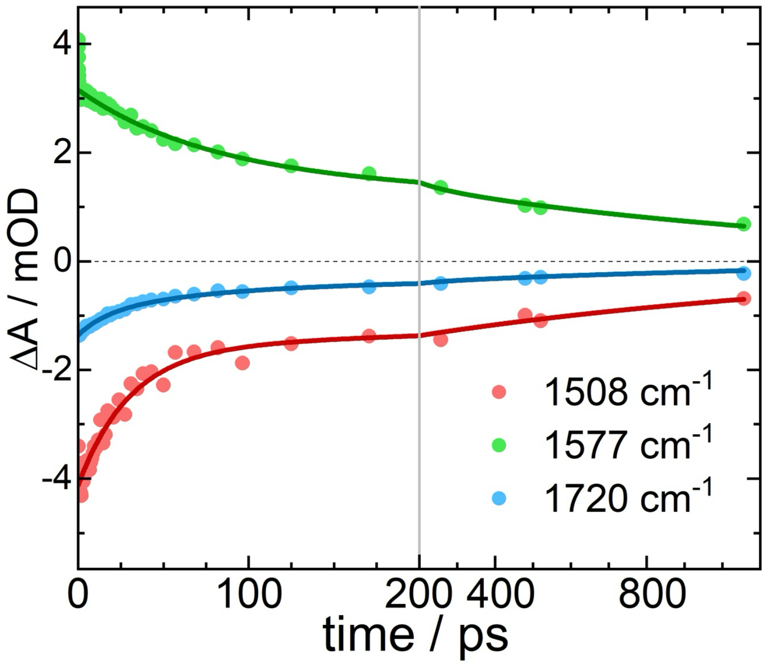

Fig. S63 Time traces for various bands of the transient IR difference spectra of the donor-acceptor cage $[Pd_2\mathbf{D1}_2\mathbf{A2}_2]^{4+}$ in CH_3CN after 400 nm excitation (note that the time axis scale changes at 200 ps).

8. References

[33] M. Frank, J. Ahrens, I. Bejenke, M. Krick, D. Schwarzer, G. H. Clever, *J. Am. Chem. Soc.* **2016**, *138*, 8279.

[49] J. Tessarolo, H. Lee, E. Sakuda, K. Umakoshi, G. H. Clever, *J. Am. Chem. Soc.* **2021**, *143*, 6339.

[54] M. Frank, J. Hey, I. Balcioglu, Y. Chen, D. Stalke, T. Suenobu, S. Fukuzumi, H. Frauendorf, G. H. Clever, *Angew. Chem. Int. Ed.* **2013**, *52*, 10102.

[61] M. U. Munshi, G. Berden, J. Martens, J. Oomens, *Phys. Chem. Chem. Phys.* **2017**, *19*, 19881.

[64] A. Burkhardt, T. Pakendorf, B. Reime, J. Meyer, P. Fischer, N. Stübe, S. Panneerselvam, O. Lorbeer, K. Stachnik, M. Warmer, P. Rödig, D. Göries, A. Meents, *Eur. Phys. J. Plus* **2016**, *131*, 56.

[65] W. Kabsch, *Acta Crystallogr. D Biol. Crystallogr.* **2010**, *66*, 125.

[66] G. M. Sheldrick, *Acta Crystallogr Sec. A* **2015**, *71*, 3.

[67] G. M. Sheldrick, *Acta Crystallogr Sec. C* **2015**, *71*, 3.

[68] C. B. Hübschle, G. M. Sheldrick, B. Dittrich, *J. Appl. Crystallogr.* **2011**, *44*, 1281.

[69] A. L. Spek, *Acta Crystallogr. Sect. C* **2015**, *71*, 9.

[70] A. L. Spek, *Acta Crystallogr. Sect. D* **2009**, *65*, 148.

[71] A. Thorn, B. Dittrich, G. M. Sheldrick, *Acta Crystallogr. Sect. A* **2012**, *68*, 448.

[72] D. Rehm, A. Weller, *Isr. J. Chem.* **1970**, *8*, 259.

[73] Wavefunction, Inc., Spartan`18 Parallel Suite, Irvine (USA), **2018**.

[74] M. J. Frisch, G. W. Trucks, H. B. Schlegel et al. Gaussian 16, Revision C.01, Gaussian, Inc., Wallingford CT, 2016.

[75] Zhurko, G. and Zhurko, D. Chemcraft Graphical Program for Visualization of Computed Results.http://www.chemcraftprog.com/, **2015**.

[76] M. J. Frisch, G. W. Trucks, H. B. Schlegel et al. Gaussian 16, Revision B.01, Gaussian, Inc., Wallingford CT, 2016.

[77] J. Franz, M. Oelschlegel, J. P. Zobel, S.-A. Hua, J.-H. Borter, L. Schmid, G. Morselli, O. S. Wenger, D. Schwarzer, F. Meyer, L. González, *J. Am. Chem. Soc.* **2024**, *146*, 11272.
